# Molecular Clip Strategy of Modified Sulfur Cathodes for High‐Performance Potassium Sulfur Batteries

**DOI:** 10.1002/advs.202405457

**Published:** 2025-01-13

**Authors:** Tianyu Chen, Zhiwen Min, Zhenjiang Yu, Mengting Zheng, Qingbin Jiang, Huifang Xu, Yuanmiao Sun, Kwan San Hui, Chenyang Zha, Jun Lu, Kwun Nam Hui

**Affiliations:** ^1^ Joint Key Laboratory of the Ministry of Education Institute of Applied Physics and Materials Engineering University of Macau, Avenida da Universidade Taipa Macau SAR 999078 China; ^2^ College of Chemical and Biological Engineering Zhejiang University Hangzhou 310027 China; ^3^ Institute of Technology for Carbon Neutrality Shenzhen Institute of Advanced Technology Chinese Academy of Sciences Shenzhen 518055 China; ^4^ Department of Mechanical Engineering College of Engineering Prince Mohammad Bin Fahd University P.O. Box 1664 Al Khobar 31952 Kingdom of Saudi Arabia

**Keywords:** chain‐like S_6_, molecular clip, potassium sulfur batteries

## Abstract

Potassium‐sulfur (K‐S) batteries are severely limited by the sluggish reaction kinetics of the cyclooctasulfur (cyclo‐S_8_) electrode with low conductivity, which urgently requires a novel cathode to facilitate activity to improve sulfur utilization. In this study, using the wet chemistry method, the molecular clip of Li^+^ is created to replace cyclo‐S_8_ molecular with the highly active chain‐like S_6_^2−^ molecular. The molecular clip strategy effectively lowers the reaction barrier in potassium‐sulfur systems, and the stretching of S─S bonds weakens the binding between sulfur atoms, facilitating the transformation of potassium polysulfides (KPSs). The as‐prepared cathode exhibits a reversible capacity of 894.8 mAh g^−1^ at a current rate of 0.5 C. It maintains a long cycle life of 1000 cycles with a stable coulombic efficiency in the potassium–sulfur cells without cathode catalysts. Operando XRD and Raman spectra combined with density functional theory (DFT) calculations, revealing the high efficiency of enhanced conversion of potassium polysulfide for high‐performance K‐S batteries.

## Graphical abstract

A molecular clip strategy is employed to design a novel electrode for potassium‐sulfur batteries, replacing the traditional ring structure S₈ with the chain‐like S₆^2^−. This approach significantly reduces the reaction barrier and enhances the conductivity compared to conventional S₈ cathode. The elongation of S─S bonds in S₆^2^^−^ weakens interactions of sulfur atoms, thereby promoting the efficient conversion of potassium polysulfides.

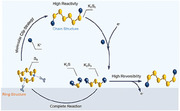

## 1 Introduction

Potassium‐sulfur (K‐S) battery technologies attract massive and substantial studies due to their outstanding potential performance involving a large theoretical specific capacity of 1675 mAh g^−1^, a high energy density, and an appropriate operating voltage range.^[^ ^1^, ^2^, ^3^, ^4^ ^]^ However, the sluggish electrochemical reaction pathway and redox kinetics in K‐S batteries are caused by the passivated cyclooctasulfur (cyclo‐S_8_), which has poor conductivity and redox reactivity.^[^ ^5^, ^6^ ^]^ Some studies have enhanced the conductivity of sulfur electrodes by loading sulfur onto materials with a large specific surface area, such as mesoporous CMK‐3^[^ ^7^ ^]^ or carbon nanotubes (CNTs).^[^ ^8^ ^]^ However, in the absence of catalysts, these electrodes only provide a capacity of 512.7–585 mAh g^−1^ under low current conditions, significantly below the theoretical capacity of K‐S batteries. Some pioneering efforts appending catalysts to enhance the electrochemical activity of K‐S system, such as the composite of tungsten single atom and tungsten carbide (W_SA_‐W_2_C@NC), single‐atom Co‐anchored between two alkynyls of graphdiyne (Co‐GDY) and sulfur host containing Cu single atoms (Cu‐N_4_).^[^ ^9^, ^10^, ^11^ ^]^ These catalysts can effectively promote the rapid conversion of potassium polysulfides. However, few studies have reported improving the slow electrochemical reaction process by modifying the cyclooctasulfur (cyclo‐S_8_）.

Addressing these issues of sulfur material necessitates a strategic approach, particularly in the structural design of sulfur molecules, which includes optimizing their spatial size and conformation.^[^ ^12^, ^13^, ^14^ ^]^ Recent studies have demonstrated that modifying the structure of materials can significantly enhance the sluggish reaction kinetics of cyclo‐S_8_ by altering the occupancy of electron orbitals in sulfur molecules, thereby affecting their electrophilic ability and conductivity.^[^ ^15^, ^16^, ^17^ ^]^ Chen et al. conducted density functional theory (DFT) calculations to investigate electron orbital energies and found that Li_2_S_8_ exhibits superior electron‐accepting properties and higher participation in electrochemical reactions than S_8_.^[^ ^18^ ^]^ Similarly, Miao et al.^[^ ^17^ ^]^ proposed a novel approach for synthesizing short‐chain sulfur molecules (S_2‐4_) using the space‐limited domain principle. These short‐chain sulfur molecules were utilized as cathodes in aqueous Cu‐S batteries, resulting in an impressive reversible capacity of 3133 mAh g^−1^ with an initial Coulombic efficiency of 96%. Their study revealed that these short‐chain sulfur molecules possess higher electron affinity energy and Fukui index, indicating enhanced reactivity.

In the realm of K‐S battery performance enhancement, notable breakthroughs have been achieved through the strategic refinement of sulfur's molecular and spatial structures, which have substantially improved electrochemical reaction kinetics, including single‐atom sulfur,^[^ ^19^ ^]^ short‐chain sulfides^[^ ^20^ ^]^ and organosulfur compounds.^[^ ^21^, ^22^, ^23^ ^]^ Xiong et al. thermally treated cyclo‐S_8_ at 600 °C, achieving a downsized molecular form (S_2_‐S_3_) and concurrently enhancing the sulfur electrode's conductivity by amalgamating a microporous carbon matrix, leading to a commendable reversible capacity. At 20 mA g^−1^, a high reversible capacity of 1198.3 mA h g^−1^ was achieved due to the higher free energy of the amorphous slight molecule sulfur in the K‐S battery.^[^ ^20^ ^]^ In addition, the sulfurized polyacrylonitrile (SPAN) was applied in the K‐S battery, where the sulfur is covalently anchored in PAN backbones in the form of short sulfur chains with C‐S bonds.^[^ ^24^ ^]^ Moreover, Yang K. et al. designed a hierarchical structure K‐S cathode based on SPAN, with areal capacities of 3.1 and 4.2 mAh cm^−2^ achieved even at high sulfur loadings of 3 and 7 mg cm^−2^.^[^ ^25^ ^]^ These modified sulfur materials have greatly improved the performance of K‐S batteries. Unfortunately, the abovementioned methods required high‐temperature treatment or complex chemical processes, limiting industrial applications. For facilitating the widespread commercialization of K‐S cells, the straightforward and ingenious structural design methods of sulfur material are of utmost importance in enhancing the performance of K‐S batteries in practical applications.

To this end, we report the preparation of a chain molecule‐based sulfur cathode and its application for potassium‐ion storage. In the cropping of bulk S_8_, the prepared lithium ions are used as molecular scissors to cleave the S_8_ ring into a chain structure S_6_^2−^ by the facile solid dissolution processes. Acting as “precursor” activated materials, the Li_2_S_6_ can be automatically converted into potassium polysulfide during the first discharging process. This tailored structure of S_6_^2−^ can reduce the high reaction barrier of cyclo‐S_8_ by lowering the sulfur molecule's lowest unoccupied molecular orbital (LUMO), making it easier to undergo nucleation reactions. Besides, a shortened band gap enhances the molecule's conductivity and electron transfer rate. Furthermore, after clipped, the S─S bonds are elongated, reducing the interatomic forces between sulfur atoms, which enhances the dissociation of polysulfides. This modification in molecular structure contributes to improved electrochemical performance by facilitating more efficient sulfur utilization and mitigating the challenges associated with polysulfide shuttling. By the unique molecular structure design, the K‐S cell achieved a remarkable reversible specific capacity of 1326.6 and 894.8 mAh g^−1^ at specific currents of 0.2 C and 0.5 C, respectively, while maintaining stability over 1000 cycles. More importantly, this molecular structure modification strategy can provide valuable insights for the rational design of highly reactive electrode materials.

## 2 Results and Discussion

### 2.1 Material Characterizations

**Figure 1:**
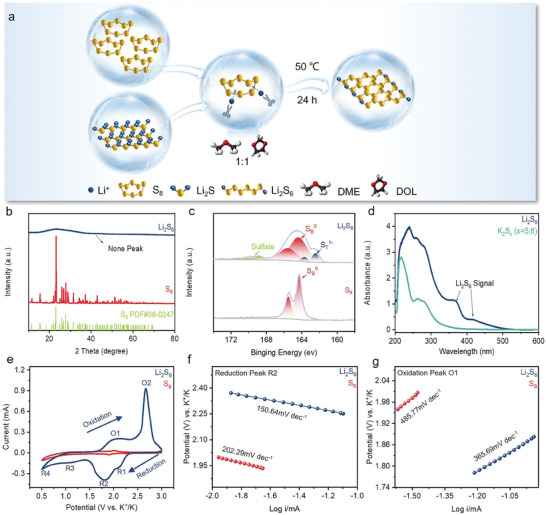
a) Synthesis schematic of Li_2_S_6_ catholyte; Characterization differences of Li_2_S_6_ and S_8_: b) XRD patterns of Li_2_S_6_ and S_8_. c) High‐resolution XPS spectra of Li_2_S_6_ and S_8_. d) UV–vis absorption spectra of Li_2_S_6_ and initial discharge to 2 V. e) CV profiles of Li_2_S_6_ and S_8_‐based cells at a scan rate of 0.1 mV s^−1^ and corresponding Tafel plots of f) reduction and g) oxidation peak.

S_6_^2−^ was synthesized through a process involving the mixing of sulfur and lithium sulfides (Li_2_S) at a molar ratio of 5:1. The reaction mixture was prepared in a solvent consisting of DOL (1,3‐dioxolane) and DME (dimethyl ether) in a volume ratio of 1:1. The resulting mixture was vigorously stirred using a magnetic stirrer at a temperature of 50 °C for 24 h (**Figure** **1**a). This material synthesis approach is relatively simple, not requiring complex equipment or high temperatures, making it easily applicable in practical settings. Li_2_S_6_ is examined in an air‐isolated ex situ X‐ray diffraction (XRD) analysis after dried organic solvent, as shown in Figure 1b. Upon comparing the XRD patterns, it is observed that the Li_2_S_6_ does not exhibit obvious characteristic peaks. However, S_8_ demonstrates distinct signal peaks (Figure 1b). The amorphous structure of Li_2_S_6_ may account for this enhancement in reactivity by the more uniform distribution on the electrode. Differences in molecular properties of Li_2_S_6_ and S_8_ were further analyzed by the ex situ S 2p X‐ray photoelectron spectroscopy (XPS) (Figure 1c). S_8_ has only bridging sulfur (164.30 eV of S 2p_3/2_), confirming the ring structure. In contrast, the Li_2_S_6_ has two peaks at 162.37 and 164.31 eV ascribed to the S 2p_3/2_ signals of terminal (S_T_^1−^) and bridging (S_B_^0^) sulfur, respectively, causing the presence of these peaks to be indicative of a chain structure. The disparities in structural configurations dictate distinct electron orbital occupancy energy levels, consequently endowing S_6_^2−^ with enhanced electrochemical activity.

The battery discharged to 2 V was disassembled, and the cathode was immersed in a DME solution for UV–vis absorption spectroscopic analyses. The results were compared with those of the Li_2_S_6_ solution (Figure 1d), revealing that they exhibit distinct characteristic peaks. Li_2_S_6_ can automatically transform into K_2_S*_x_* (*x* = 5,6) during the first cycle of charging and discharging. Figure 1e presents the cyclic voltammetry (CV) curves of the cells with Li_2_S_6_ and S_8_ electrodes, spanning a voltage window of 0.5–3 V. The Li_2_S_6_ electrode demonstrates a pronounced redox peak, whereas the S_8_ electrode manifests a more subdued curve devoid of any significant reaction peaks. This observation underscores the profound impact of molecular structural variations on redox reactivity during the charging and discharging processes. For the Li_2_S_6_ electrode, the initial electrochemical cycle revealed the presence of two distinct cathodic peaks, occurring at potentials of 2.08 V (R1) and 1.81 V (R2), as well as two broader cathodic features manifested at 1.06 V (R3) and 0.50 V (R4). These discernible regions within the voltammetric profile correspond to the electrochemical processes associated with the formation of long‐chain polysulfides (K_2_S_4—6_) and short‐chain polysulfides (K_2_S_1–3_), respectively. Notably, the subsequent electrochemical behavior exhibited two anodic peaks, observed at potentials of 2.04 V (O1) and 2.67 V (O2). Significantly, the ensuing electrochemical behavior displayed two anodic peaks, observed at potentials of 2.04 V (referred to as O1) and 2.67 V (designated as O2), respectively, representing the depotassiation reactions of solid‐state polysulfides (K_2_S_1–3_) and soluble polysulfides (K_2_S_4–6_).^[^ ^26^ ^]^ Excluding the salient peak at 2.67 V, the CV peaks exhibit a relatively even topography, suggesting that the electrochemical reactions within these voltage spectrums are multifaceted. This can be attributed to the equilibrium phase of potassium polysulfides (KPSs) in the redox procedure being considerably more intricate than lithium polysulfides (LiPSs). It is conceivable that multiple KPSs coexist at identical voltage.^[^ ^2^ ^]^

Notably, during the first five cycles, the CV curves of the cell display remarkable congruence, signifying the commendable reversibility of the redox reactions in the cell (Figure S1, Supporting Information). In stark comparison, the S_8_ exhibits only three faint peaks (corresponding R2, R3, and O1), indicative of its languid kinetics and uncompleted reaction. The presence of a single peak in the voltage range of 1.5–0.5 V indicates an incomplete conversion to solid‐state polysulfides. Simultaneously, the dissolution efficiency of solid‐state polysulfides during the oxidation process is also relatively low. The suboptimal reactivity of S_8_ consequently results in a non‐uniform SEI. An in‐depth analysis of the Tafel slopes throughout the oxidation and reduction phases of S_8_ and Li_2_S_6_ is shown in Figure 1f,g. Additional data for other peaks can be found in Figure S2 (Supporting Information). Li_2_S_6_ consistently possesses more diminutive slope values, indicative of an accelerated reaction rate.

### 2.2 Electrochemical Performance

**Figure 2:**
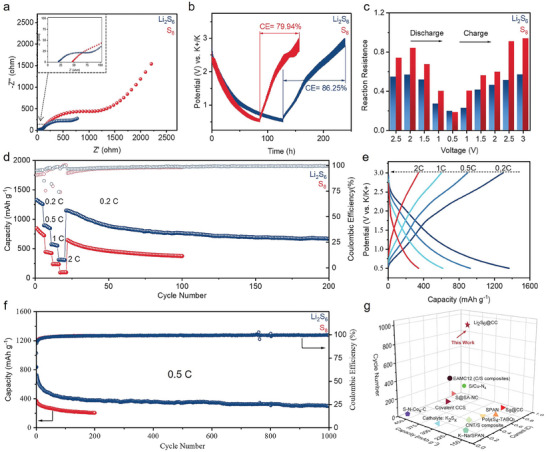
Electrochemical properties of K‐S cells. a) Nyquist plots, b) GITT discharge–charge voltage profiles, c) the calculated in situ reaction resistance of Li_2_S_6_@CC and S_8_@CC from GITT, d) rate performance under different rates, e) galvanostatic charge/discharge profiles under different rates, f) long‐term cycle performance at 0.5 C of Li_2_S_6_@CC and S_8_@CC K‐S cells and g) cell performance comparison of recently reported work in K‐S cells.

The performance of Li_2_S_6_ and S_8_ was evaluated via electrochemical analysis to delineate their respective electrochemical traits. To ensure uniformity of comparison, the Li_2_S_6_ and S_8_ batteries are made of the same carbon cloth current collector (This electrode is used to facilitate the loading of Li_2_S_6_, the electrochemical test of that is shown in Figure S3, Supporting Information). **Figure** **2**a visually presents the electrochemical impedance spectroscopy (EIS) spectra corresponding to both cells. Upon contrasting the S_8_‐based cell with the Li_2_S_6_‐based cell, it is discernible that the latter demonstrates a reduced charge transfer resistance (R_ct_) within the high‐frequency domain of the Nyquist plots. Additionally, the variation in the resistance of the battery after 200, 500, and 1000 cycles, along with the corresponding fitting results, is presented in Figure S4 (Supporting Information). The interfacial contact resistance (R_ins_) stabilizes around a constant value, suggesting the presence of a stable interface and proficient electron transfer for Li_2_S_6_ electrode, even after 1000 cycles. The fitting results in Figure S5 (Supporting Information) show that R_ins_ keeps stable at ≈700 Ω. This indicated a stable interface and effective charge transfer remained in the Li_2_S_6_ electrode even after long cycling. These findings indicate superior conductivity and stable cathode electrolyte interface (CEI) improved electrochemical kinetics in the Li_2_S_6_‐based cell.

In the context of the galvanostatic intermittent titration technique (GITT) procedure, as depicted in Figure 2b, the protocol is structured such that a discharge pulse persists for a span of 5 min, succeeded by a relaxation interval of 1 h, during which no current permeates the cell. A GITT charge process ensues, comprising a 5 min charge pulse, followed by a subsequent relaxation duration of 1 h. Within this experimental paradigm, the Li_2_S_6_‐based K‐S battery outperforms its S_8_ counterpart, as evidenced by its superior coulombic efficiency, enhanced capacity, and reduced overpotential; the locally amplified part of the final charge stage clearly shows the difference of overpotential (Figure S6, Supporting Information). Based on the GITT curve (Formula S1, Supporting Information), the differences in reaction resistance were computed (Figure 2c). The results indicate that, except for discharge to the lowest voltage of 0.5 V, Li_2_S_6_ exhibits a minor kinetic barrier compared to S_8_ throughout the charge–discharge process.^[^ ^27^, ^28^ ^]^ The sluggish kinetics of S_8_ are attributed to its cyclic molecular structure and lower conductivity.

Symmetric cells are employed to further probe the activity and stability of the Li_2_S_6_ and S_8_‐based batteries. The symmetric battery, characterized by identical sulfur electrodes at the cathode and anode, facilitates the comparison of electrochemical performance between the two materials. As illustrated in the cyclic voltammetry (CV) curves (Figure S7, Supporting Information), the Li_2_S_6_ symmetric cell exhibits a heightened current and a multiplicity of potential responses compared to the S_8_ electrode, a testament to the enhanced electrochemical activity inherent in Li_2_S_6_.^[^ ^29^ ^]^ To exclude the influence of Li^+^, a symmetrical cell was constructed using the commercial electrolyte of lithium‐sulfur batteries. The symmetric cell exhibited different peak positions (Figure S8a, Supporting Information), demonstrating that K‐S batteries operate on a reaction mechanism distinct from Li‐S batteries. Additionally, symmetrical cells were tested with the electrolyte corresponding to Figure S8 (Supporting Information) and without KTFSI (Figure S8b, Supporting Information). The absence of a stable peak formation confirmed that the results in Figure S8 (Supporting Information) arise from the reaction of potassium polysulfides.

The rate performance of both materials is scrutinized under multiple current densities. Figure 2d presents a compelling demonstration of the Li_2_S_6_’s advantageous discharge capacities at varying current densities, registering 1326.6, 894.8, 571.8, and 310.5 mAh g^−1^ at 0.2 C, 0.5 C, 1 C, and 2 C, respectively (1 C = 1675 mA g^−1^). Notably, even after functioning at a high current density of 2 C, the capacity effectively recuperates once the current rate reverts to 0.2 C. Furthermore, the Li_2_S_6_ electrode upholds the fidelity of charge and discharge curves even under the stringent conditions of a 2 C current density, underscoring its exceptional fast‐charging prowess and structural durability (Figure 2e). In contrast to the Li_2_S_6_ electrode, the S_8_ electrode encounters only 79.3 mAh g^−1^ at a current density of 2 C, a consequence attributable to the heterogeneity of the electrochemical reactions. The performance of Li_2_S_6_ is further exemplified by its impressive high capacity of 294 mAh g^−1^, even after subjecting it to 1000 continuous cycles under 0.5 C. This commendable feat is complemented by an impressively minimal fading rate of 0.059% per cycle (Figure 2f), thereby underscoring the extraordinary performance of Li_2_S_6_. For higher sulfur load and less E/S ratio, the performance data is shown on Figure S9 (Supporting Information), this proves the high energy density for practical application. Furthermore, graphite was prepared as the counter electrode to investigate whether lithium ions contribute to the capacity, with the Li_2_S_6_ electrode serving as the positive electrode. The Li‐ion cell exhibited a specific capacity of 10 mAh g^−1^, underscoring the small capacity contribution provided by lithium ions in this system (Figure S10, Supporting Information). As Figure 2g vividly illustrates, this result transcends the S_8_ cell and the lion's share of extant K‐S batteries without catalysts reported in the literature. Also, a detailed data comparison is shown in Table S1 (Supporting Information).

### 2.3 The Uniformities of Volumetric Expansion of Li_2_S_6_ Electrode

Beyond the notable impact of molecular structure on reactivity, the exceptional cycle stability and reversible capacity of the Li_2_S_6_ electrode can be attributed to uniform volume expansion. It is imperative to note that potassium atoms, possessing a larger atomic radius compared to lithium (Li 0.68 Å, K 1.38 Å), precipitate an expansive volumetric escalation upon their intimate interaction with sulfur atoms.^[^ ^30^ ^]^ This volumetric augmentation predominantly accounts for the impediment in realizing protracted cycles in numerous potassium‐sulfur systems.

**Figure 3:**
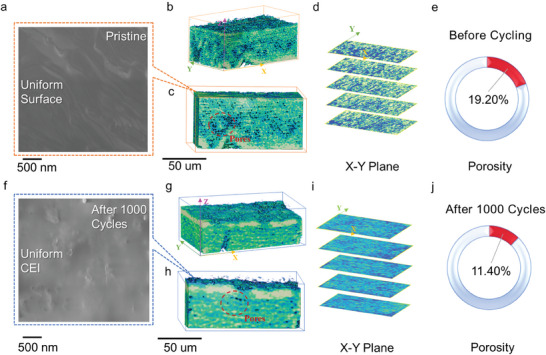
SEM images of electrode surfaces: a) pristine and f) after 1000 cycles of Li_2_S_6_ electrode; 3D morphology of pristine and after 1000 cycles of Li_2_S_6_ electrode conducted by Computed Tomography: b) Pristine Li_2_S_6_ electrode slice morphology; c) *X‐Z* plane; d) Slice the X‐Y plane along the *Z* axis; e) Porosity of the electrode. g) After 1000 cycles, Li_2_S_6_@CC slice morphology; h) *X‐Z* plane; i) Slice the *X‐Y* plane along the *Z* axis; j) electrode porosity.

Scanning electron microscope (SEM) technology is used to observe the surface of the electrode difference between Li_2_S_6_ and S_8_. SEM imagery of the pre‐cycled Li_2_S_6_ electrode (**Figure** **3**a) reveals a homogeneous dispersion of liquid Li_2_S_6_ across the carbon cloth surface after a dropwise application. The Energy Dispersive X‐ray Spectroscopy (EDX) sulfur element mapping, as illustrated in Figures S9 and S10 (Supporting Information), provides corroborative substantiation of the consistent surface distribution of Li_2_S_6_ electrode sulfur species (Figure S11a, Supporting Information), persisting even upon completion of 1000 cycles (Figure S11b, Supporting Information). In contrast, the S_8_ electrode's surface distribution appears a little uneven (Figure S12a, Supporting Information), with a correspondingly diminished sulfur signal following cycling (Figure S12b, Supporting Information). The homogeneous surface of the Li_2_S_6_ electrode is beneficial in accommodating volume expansion issues during charge and discharge cycles, thereby enabling the formation of a stable and smooth cathode electrolyte interphase (CEI). Impressively, even after 1000 cycles, the CEI of Li_2_S_6_ retains relative flatness and smoothness (Figure 3f). In stark contrast, the S_8_ electrode manifests an aggregated state on its surface (refer to Figure S13a, Supporting Information), a condition instigated by the binder. Consequently, this leads to the creation of disproportionate extreme volume expansion stress when coupled with potassium ions. This imbalanced stress results in significant cracking at the CEI interface post‐cycling (Figure S13b, Supporting Information), ultimately leading to premature termination of the battery's life cycle.

In pursuit of an in‐depth comprehension of the Li_2_S_6_ electrode's accommodation to such volumetric dynamics, we embarked on a meticulous 3D structural assessment of the Li_2_S_6_ electrode at their pristine state and following 1000 cycles, employing Computed Tomography (CT) scanning.^[^ ^31^ ^]^ Figure 3b delineates the pristine Li_2_S_6_ electrode slice morphology, which intriguingly retains its porous architecture post‐uniform Li_2_S_6_ sorption. The X‐Z plane (Figure 3c) further elucidates the extensive porosity, underscoring the electrode's potential to mitigate potassium polysulfide's volume expansion during cycling. From a quantitative perspective, the electrode was dissected orthogonally to the Z‐axis, revealing the X‐Y plane (Figure 3d). The plane's defects, manifested in a distinct blue hue, serve as the foundation for calculating the proportional porosity across individual planes, culminating in an average initial porosity value of 19.2% (Figure 3e). Furthermore, following the adsorption of Li_2_S_6_, the porosity remains relatively uniformly distributed, with no extensive blockage observed. This observation provides evidence of the spontaneous and uniform adsorption of Li_2_S_6_ onto the carbon cloth electrode. Post 1000 cycles, analysis shows minimal deformation in the electrode (Figure 3g), with a relatively uniform SEI on its surface. The lateral X‐Z plane (Figure 3h) vividly brings forth the extant porosity. Post‐cycling, while porosity becomes partially replete with volume expansion, the distribution persists relatively uniformly (Figure 3i). This phenomenon underscores the beneficial role of uniform adsorption in facilitating the accommodation of significant volume expansion during the cycling process in carbon cloth electrodes. Consequent calculations yield a post‐cycling porosity ≈11.4% (Figure 3j). This attenuated porosity indicates some irreversible deformation transpiring over the cycles, a phenomenon that resonates with prior findings.^[^ ^3^ ^]^ Nevertheless, the residual 11.4% porosity serves as a testament to the Li_2_S_6_ electrode's formidable resilience in bracing the pronounced volumetric expansion endemic to potassium‐sulfur batteries, thereby safeguarding its structural integrity. This provides a reliable idea for designing long‐cycle high‐performance K‐S batteries.

### 2.4 Mechanism Analysis

For the optimization and longevity enhancement of potassium‐sulfur batteries, elucidating the intricacies of material conversion during both the charging and discharging phases remains paramount. Utilizing *operando* X‐ray diffraction (XRD) and Raman techniques, the present investigation pioneers exploring the electrochemical dynamics of K‐S transformations in ether‐based electrolytes.

**Figure 4:**
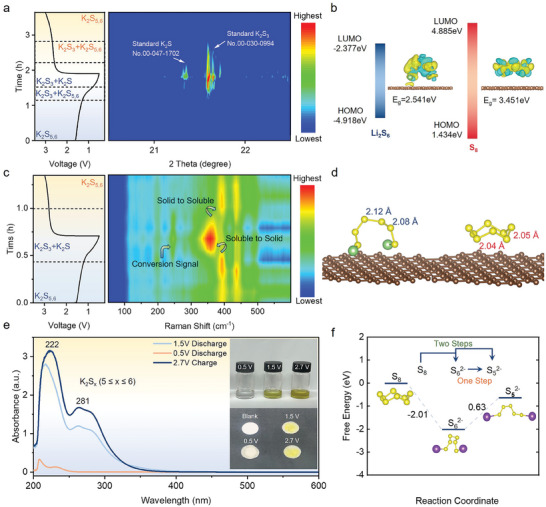
Galvanostatic discharge, charge curve, and corresponding a) *operando* XRD spectra and c) *operando* Raman for Li_2_S_6_ cathode. DFT calculated the lowest unoccupied molecular orbital (LUMO) and highest occupied molecular orbital (HOMO) of b) Li_2_S_6_ and S_8_ with charge density difference pattern on the right_,_ d) and the bond length of S─S of Li_2_S_6_ and S_8._ e) UV–vis absorption spectra of the polysulfides solutions. Insets show optical images of the solution and separator of 2.7 V (charging), 1.5 V (discharging), and 0.5 V (full discharging). f) The change processes of Gibbs free energy from S_8_ to S_5_^2−^.

**Figure** **4**a shows the result of the Li_2_S_6_‐based K‐S battery; at the discharge to 1.35 V, the diffraction peak signal appeared at 21.65° and is assigned to solid K_2_S_3_ (PDF No. 00‐030‐0994). Also, at the discharge to 0.87 V, the diffraction peak signal appeared at 21.3°. that is assigned to solid K_2_S (PDF No. 00‐047‐1702). The original data results are shown in Figure S14 (Supporting Information).^[^ ^32^ ^]^ This spectrum denotes the electrochemical transformation of the soluble species K_2_S*_x_* (*x* = 5, 6), predominantly transitioning to K_2_S_3_, with a lesser extent evolving to K_2_S on the electrode's surface. The K_2_S_3_ peak intensifies commensurate with an increased discharge depth, ultimately suggesting a discharge product comprised predominantly of K_2_S_3_ and K_2_S constituents. There have been previous papers reporting such discharge products.^[^ ^33^ ^]^ The subsequent charging process delineates the reversible electrochemical behavior of the solid potassium polysulfides as they re‐convert to their soluble K_2_S*_x_* forms, displayed by the progressive attenuation of respective XRD signals. The signals corresponding to K_2_S and K_2_S_3_ are observed to fully vanish at 2.55 and 2.7 V, respectively. This observation suggests that the solid‐state potassium polysulfides can undergo a completely reversible transformation during the charging process, which is likely a contributing factor to the extended cycling stability of the battery. Strategically structured molecular configurations have been instrumental in counteracting inherent dead sulfur limitations of the K‐S battery. When fully charged to 3.0 V, the electrode was analyzed by ex situ XPS test (Figure S15, Supporting Information). At this time, the morphology of the S element was S_8_ and potassium polysulfide. This result has also been reported in other papers.^[^ ^32^, ^34^ ^]^ Conversely, Figure S16 (Supporting Information) showcases the S_8_‐based K‐S battery spectrum, conspicuously absent of prominent XRD signal peaks. The original data results are shown in Figure S17 (Supporting Information). This data underscores the reactivity limitations of the cyclic S_8_ molecule, where suboptimal sulfur utilization coupled with inherent resistive barriers impedes reaction completion, resulting in diminished electrochemical capacity and operational longevity. The S_8_ battery could not observe the signal of K_2_S_3_ and K_2_S mainly because the preconversion reaction step was blocked. According to *Le Chatelier's principle*, the conversion rate of S_8_ to potassium polysulfide will greatly affect the output of the final discharge products K_2_S_3_ and K_2_S, resulting in signals that are too weak to be detected. The relevant mechanism has been reported in other literature.^[^ ^35^ ^]^

*Operando* Raman testing also confirms the clear transformation process of polysulfides (Figure 4c), with long‐chain polysulfides K_2_S*_x_* (*x* = 5,6) exhibiting peaks at 390 and 440 cm^−1^ at the early stages of discharge. Upon discharging to 1.35 V, a shift in peak positions occurs, concentrating at 360 cm^−1^, with the original Raman peaks disappearing and new peaks emerging at 245 cm^−1^. This shift corresponds to the XRD results during discharge, indicating the formation of solid K_2_S_3_ and K_2_S. During the charging process, the Raman peaks reversibly transform back to soluble polysulfides. The original data is presented in Figure S18 (Supporting Information). In contrast, the *operando* Raman spectra of cyclic S_8_ show distinct peaks at 280, 310, 325, and 410 cm^−1^ (Figure S19, Supporting Information). During the battery cycling process, the signals weaken but do not completely disappear or produce new peaks. This indicates that solid cyclic S_8_ cannot fully combine with potassium ions due to a prohibitively large reaction barrier. The original data can be found in Figure S20 (Supporting Information).

The computational results obtained through density functional theory (DFT) calculations of the lowest unoccupied molecular orbital (LUMO) and highest occupied molecular orbital (HOMO) of Li_2_S_6_ and S_8_ also elucidate the enhanced reactivity of S_6_^2−^ (Figure 4b). Notably, Li_2_S_6_ exhibits a markedly lower LUMO energy level (−2.377 eV) in comparison to S_8_ (4.885 eV). This reduced LUMO energy signifies a heightened electron affinity, indicating that Li_2_S_6_ is more likely to accept electrons and convert into short‐chain polysulfides.^[^ ^36^ ^]^ Moreover, the band gap (E_g_) of Li_2_S_6_ is smaller than S_8_ (2.541 eV vs. 3.451 eV), showing enhanced electrical conductivity and swifter electron transfer rates. Consequently, the Li_2_S_6_ electrode displays lower electrical resistance.^[^ ^37^ ^]^ The charge density difference results on the right indicate that the interaction between Li_2_S_6_ and the carbon substrate is notably stronger than with S_8_, underscoring the enhanced electron transfer capabilities of the Li_2_S_6_ electrode. These findings collectively underpin the excellent reversibility and high specific capacity exhibited by the Li_2_S_6_ electrode. Besides, the heightened reactivity of clipped Li_2_S_6_ molecules is also attributed to changes in the interatomic forces between sulfur atoms. DFT calculations reveal that in Li_2_S_6_ molecules, all the S─S bonds are elongated, with the longest reaching 2.12 Å, compared to 2.04 or 2.05 Å in S_8_ molecules (Figure 4d). The elongation of S─S bonds facilitates the cleavage of these bonds to form solid K_2_S_3_/K_2_S, thereby enhancing reaction reactivity and the utilization of active materials. Furthermore, the enhanced efficiency of the Li_2_S_6_ reaction also stems from a more direct reaction pathway. Compared to the two energy conversion pathways required for the transformation of S_8_ to S_5_^2−^, the reduction of S_6_^2−^ minimizes the solid‐to‐liquid phase transition, allowing for the efficient direct conversion to S_5_^2−^ on the current collector (Figure 4f).

These findings were corroborated by UV–vis absorption spectroscopic analyses (Figure 4e). The batteries at this voltage were disassembled, and the sulfur electrode was extracted and immersed in DME solvent. The supernatant was diluted and subjected to UV–vis absorption spectroscopic testing (Figure S21, Supporting Information). Electrolytic solutions under various potential biases, specifically 1.5 and 2.7 V, manifested a deep brown hue, revealing UV absorption signatures at 222 and 281 nm, respectively, under 1.5 and 2.7 V biases. In stark contrast, the electrolyte post exhaustive discharge at 0.5 V displayed near transparency and close to no signal peak. These spectral signatures, emblematic of soluble polysulfide intermediates, are resonant with reported spectral data acquired from chemically synthesized K_2_S*_x_* (*x* = 5, 6) entities, implying the likely emergence of K_2_S_6_ and K_2_S_5_ species.^[^ ^1^, ^38^ ^]^ Upon intensified discharging to a potential of 0.5 V, the resultant electrolyte portrayed optical clarity, devoid of any discernible UV–vis signatures. Upon further discharge to 1.2 V, the collected solution becomes colorless, and no UV–vis band is detected. This observation suggests the further reduction of long‐chain K_2_S*_x_* (*x* = 5, 6) into insoluble short‐chain polysulfide K_2_S_3_ and K_2_S, a statement congruent with *operando* XRD and Raman outcomes.

**Figure 5:**
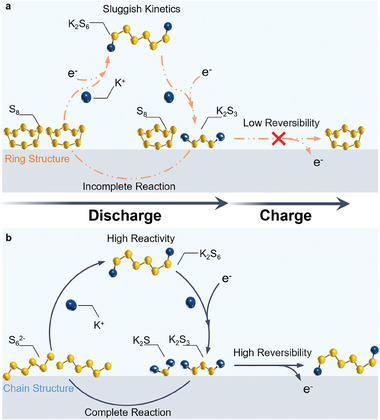
Schematic of mechanism of charge and discharge in a) S_8_ and b) Li_2_S_6_‐based K‐S batteries.

**Figure** **5** presents a schematic representation delineating the charging and discharging mechanisms of K‐S batteries utilizing both S_8_ and Li_2_S_6_ as the active material. Figure 5a outlines the inferior sulfur utilization efficiency of solid‐state cyclo‐S_8_ molecules, showing an incomplete reaction within the K‐S system. This inadequacy is ascribed to their relatively low electrical conductivity and sluggish reaction kinetics. Figure 5b illustrates that homogeneous adsorption occurs when the prepared brown Li_2_S_6_ solution is evenly dispersed on the electrode and gains high conductivity. As excess potassium ion infiltrates from the electrolyte, it gradually replaces lithium‐ion as the connecting site of S_6_^2−^ and circulates stably during the charge and discharge processes. When modified by lithium ions in a chain configuration, the molecular structure demonstrably mitigates the reaction barrier. Such an alteration facilitates more comprehensive reaction kinetics and engenders enhanced reversibility.

To empirically ascertain the practical applicability of the Li_2_S_6_‐facilitated K‐S battery system, dual button cells were harnessed to sustainably illuminate an LED with the inscription “UM2023” (Figure S22a, Supporting Information). Additionally, a singular cell effectively powers a diminutive fan (Figure S22b, Supporting Information). These empirical validations underscore the system's prospective commercial pertinence.

## 3 Conclusion

In conclusion, we have devised chain‐like S_6_^2−^ cathodes using “ion scissors” to bolster sulfur's inherently slow electrochemical reactivity in advanced K‐S batteries. On the one hand, the liquid‐state S_6_^2−^ exhibits lower reaction resistance and can achieve enhanced conductivity through uniform adsorption onto the carbon cloth substrate. On the other hand, analyses of electron energy and S─S bond length by DFT calculation and *operando* XRD and UV–vis spectroscopy confirm that the S_6_^2−^ electrode has heightened reactive activity and undergoes a complete “liquid‐solid‐liquid” conversion. Consequently, this innovative K‐S battery architecture delivers an exceptional reversible capacity of 1326.6 and 894.8 mAh g^−1^ at specific current densities of 0.2 C and 0.5 C, respectively, functioning without a catalyst. Furthermore, the cyclo‐S_8_ molecule tailoring strategy can directly guide the development of high‐performance electrochemical devices and catalysts with specialized structures. For practical application, the flexible Li_2_S_6_ cathode for K‐S batteries with arbitrary shapes could be rapidly obtained through industrial spraying on commercial carbon cloth. This cathode can be produced on a macro‐scale and features ultra‐high energy density and extremely low cost. The tailor‐designed S_6_^2−^ electrodes hold significant potential to advance the industrial application of potassium‐sulfur batteries and can also be applied in other alkali metal batteries. However, the electrochemical reaction between sulfur and potassium remains relatively slow, and the capacity decay rate is still faster than that of the more mature Li‐S batteries. This is due to K_2_S's stable thermodynamic nature, which makes it difficult to convert during the charging process fully. Therefore, it is still necessary to develop catalysts that can rapidly convert K_2_S for S_6_^2−^ based K‐S batteries, further enhancing battery performance and cycling stability.

## Conflict of Interest

The authors declare no conflict of interest.

## Author Contributions

T.C., Z.M., and Z.Y. contributed equally to this work.

## Supporting information



Supporting Information

## Data Availability

The data that support the findings of this study are available in the supplementary material of this article.

## References

[1] X. Lu , M. E. Bowden , V. L. Sprenkle , J. Liu , Adv. Mater. 2015, 27, 5915. 26305734 10.1002/adma.201502343

[2] J. Ding , H. Zhang , W. Fan , C. Zhong , W. Hu , D. Mitlin , Adv. Mater. 2020, 32, 1908007. 10.1002/adma.201908007 32249505

[3] X. Zhao , Y. Hong , M. Cheng , S. Wang , L. Zheng , J. Wang , Y. Xu , J. Mater. Chem. A 2020, 8, 10875.

[4] S. Lee , H. Park , J. Rizell , U. H. Kim , Y. Liu , X. Xu , S. Xiong , A. Matic , A. T. Zikri , H. Kang , Y. K. Sun , Adv. Funct. Mater. 2022, 32, 2209145.

[5] X. Ji , K. T. Lee , L. F. Nazar , Nat. Mater. 2009, 8, 500. 19448613 10.1038/nmat2460

[6] G. Zhou , E. Paek , G. S. Hwang , A. Manthiram , Nat. Commun. 2015, 6, 7760. 26182892 10.1038/ncomms8760 PMC4518288

[7] Q. Zhao , Y. Hu , K. Zhang , J. Chen , Inorg. Chem. 2014, 53, 9000. 25119141 10.1021/ic500919e

[8] X. Yuan , B. Zhu , J. Feng , C. Wang , X. Cai , R. Qin , J. Electron. Mater. 2021, 50, 3037.

[9] W. Song , X. Yang , T. Zhang , Z. Huang , H. Wang , J. Sun , Y. Xu , J. Ding , W. Hu , Nat. Commun. 2024, 15, 1005. 38307899 10.1038/s41467-024-45405-w PMC10837207

[10] C. Ye , J. Shan , H. Li , C.‐C. Kao , Q. Gu , S.‐Z. Qiao , Angew. Chem., Int. Ed. 2023, 62, e202301681. 10.1002/anie.202301681 36975137

[11] S. Zhang , Y. Kong , Y. Gu , R. Bai , M. Li , S. Zhao , M. Ma , Z. Li , L. Zeng , D. Qiu , Q. Zhang , M. Luo , L. Gu , Y. Yu , S. Guo , J. Zhang , J. Am. Chem. Soc. 2024, 146, 4433. 38329948 10.1021/jacs.3c09533

[12] Z. Li , H. B. Wu , X. W. Lou , Energy Environ. Sci. 2016, 9, 3061.

[13] D. Luo , G. Li , Y.‐P. Deng , Z. Zhang , J. Li , R. Liang , M. Li , Y. Jiang , W. Zhang , Y. Liu , W. Lei , A. Yu , Z. Chen , Adv. Energy Mater. 2019, 9, 1900228.

[14] Z. Min , C. Yang , G. H. Zhong , Z. Lu , ACS Appl. Mater. Interfaces 2022, 14, 18373. 35420418 10.1021/acsami.2c00292

[15] K. Shen , H. Mei , B. Li , J. Ding , S. Yang , Adv. Energy Mater. 2018, 8, 1701527.

[16] X. Li , L. Yuan , D. Liu , Z. Li , J. Chen , K. Yuan , J. Xiang , Y. Huang , Energy Storage Mater. 2020, 26, 570.

[17] Z. Miao , J. Xu , C. Xu , J. Zhang , Y. Liu , B. Wanyan , H. Yu , L. Yan , L. Zhang , J. Shu , Proc. Natl. Acad. Sci. USA 2023, 120, e2307646120. 37579150 10.1073/pnas.2307646120 PMC10450428

[18] J.‐J. Chen , R.‐M. Yuan , J.‐M. Feng , Q. Zhang , J.‐X. Huang , G. Fu , M.‐S. Zheng , B. Ren , Q.‐F. Dong , Chem. Mater. 2015, 27, 2048.

[19] G.‐Z. Yang , Y. F. Chen , B. Q. Feng , C. X. Ye , X. B. Ye , H. Jin , E. Zhou , X. Zeng , Z. L. Zheng , X. L. Chen , D. S. Bin , Energy Environ. Sci. 2023, 16, 1540.

[20] P. Xiong , X. Han , X. Zhao , P. Bai , Y. Liu , J. Sun , Y. Xu , ACS Nano 2019, 13, 2536. 30677289 10.1021/acsnano.8b09503

[21] J. Zhou , T. Qian , N. Xu , M. Wang , X. Ni , X. Liu , X. Shen , C. Yan , Adv. Mater. 2017, 29, 1701294. 10.1002/adma.201701294 28691212

[22] H. Yang , J. Chen , J. Yang , J. Wang , Angew. Chem., Int. Ed. 2020, 59, 7306. 10.1002/anie.201913540 31713966

[23] A. Bhargav , A. Manthiram , Adv. Energy Mater. 2020, 10, 2001658.

[24] Y. Liu , W. Wang , J. Wang , Y. Zhang , Y. Zhu , Y. Chen , L. Fu , Y. Wu , Chem. Commun. 2018, 54, 2288. 10.1039/c7cc09913d 29436538

[25] K. Yang , S. Kim , X. Yang , M. Cho , Y. Lee , Small Methods 2022, 6, e2100899. 35041292 10.1002/smtd.202100899

[26] K. Yang , J. Chen , S. Kim , P. Xiong , W. Chen , M. Cho , Y. Lee , ACS Energy Lett. 2023, 8, 2169.

[27] J. Zhou , X. Liu , L. Zhu , S. Niu , J. Cai , X. Zheng , J. Ye , Y. Lin , L. Zheng , Z. Zhu , D. Sun , Z. Lu , Y. Zang , Y. Wu , J. Xiao , Q. Liu , Y. Zhu , G. Wang , Y. Qian , Chem 2020, 6, 221.

[28] Q. Ren , H. Xie , M. Wang , X. Ding , J. Cui , D. Luo , C. Liu , Z. Lin , Chem. Commun. 2021, 57, 3512. 10.1039/d1cc00025j 33690759

[29] X. Zhou , X. Li , Z. Li , J. Fu , S. Xu , W. Zhou , S. Gui , L. Wei , H. Yang , J.‐F. Wu , X. Guo , Mater. Today Energy 2022, 26, 100990.

[30] E. Olsson , J. Yu , H. Zhang , H.‐M. Cheng , Q. Cai , Adv. Energy Mater. 2022, 12, 2200662.

[31] H. Michael , F. Iacoviello , T. M. M. Heenan , A. Llewellyn , J. S. Weaving , R. Jervis , D. J. L. Brett , P. R. Shearing , J. Electrochem. Soc. 2021, 168, 010507.

[32] C. Ye , J. Shan , D. Chao , P. Liang , Y. Jiao , J. Hao , Q. Gu , K. Davey , H. Wang , S.‐Z. Qiao , J. Am. Chem. Soc. 2021, 143, 16902. 34623812 10.1021/jacs.1c06255

[33] N.‐C. Lai , G. Cong , Y.‐C. Lu , J. Mater. Chem. A. 2019, 7, 20584.

[34] L. Wang , J. Bao , Q. Liu , C.‐F. Sun , Energy Storage Mater. 2019, 18, 470.

[35] H. Li , R. Meng , C. Ye , A. Tadich , W. Hua , Q. Gu , B. Johannessen , X. Chen , K. Davey , S.‐Z. Qiao , Nat. Nanotechnol. 2024, 19, 792. 38366224 10.1038/s41565-024-01614-4

[36] Z. Ye , S. Xie , Z. Cao , L. Wang , D. Xu , H. Zhang , J. Matz , P. Dong , H. Fang , J. Shen , M. Ye , Energy Storage Mater. 2021, 37, 378.

[37] Z. Tie , L. Liu , S. Deng , D. Zhao , Z. Niu , Angew. Chem., Int. Ed. 2020, 59, 4920. 10.1002/anie.201916529 31943699

[38] J.‐Y. Hwang , H. M. Kim , C. S. Yoon , Y.‐K. Sun , ACS Energy Lett. 2018, 3, 540.

