# Supplementary material for: Molecular Clip Strategy of Modified Sulfur Cathodes for High‐Performance Potassium Sulfur Batteries
Source: Adv Sci (Weinh). 2025 Jan 13;12(9):2405457. doi: 10.1002/advs.202405457 (PMC11884556; doi:10.1002/advs.202405457)
Supplement: Supplementary file 1 — Supporting Information [file ADVS-12-2405457-s001.docx]

**Supporting Information**

**Molecular Clip Strategy of Modified Sulfur Cathodes for High-Performance Potassium Sulfur Batteries**

*Tianyu Chen, Zhiwen Min, Zhenjiang Yu, Mengting Zheng, Qingbin Jiang, Huifang Xu, Kwan San Hui*, Chenyang Zha*, Jun Lu*, Kwun Nam Hui**

T. Chen, Z. Min, Z. Yu, Q. Jiang, H. Xu, Prof. C. Zha, Prof. K. N. Hui

Joint Key Laboratory of the Ministry of Education, Institute of Applied Physics and Materials Engineering, University of Macau, Avenida da Universidade, Taipa, Macau SAR, China. E-mail: [chenyangzha@um.edu.mo](mailto:chenyangzha@um.edu.mo), [bizhui@um.edu.mo](mailto:bizhui@um.edu.mo)

Prof. J. Lu, Prof. M. Zheng

College of Chemical and Biological Engineering, Zhejiang University, Hangzhou 310027, China. E-mail: [junzoelu@zju.edu.cn](mailto:junzoelu@zju.edu.cn)

Prof. K.S. Hui

Department of Mechanical Engineering, College of Engineering, Prince Mohammad Bin Fahd University, P.O. Box 1664, Al Khobar 31952, Kingdom of Saudi Arabia

E-mail: [khui@pmu.edu.sa](mailto:khui@pmu.edu.sa)

**Experimental** **Procedures**

**Fabrication of Materials**

*Synthesis of Li_2_S_6_ catholyte.* Li_2_S_6_ was synthesized through a process involving the mixing of sulfur and lithium sulfides (Li_2_S) at a molar ratio of 5:1. The reaction mixture was prepared in a solvent consisting of DOL (1,3-dioxolane) and DME (dimethyl ether) in a volume ratio of 1:1. The resulting mixture was vigorously stirred using a magnetic stirrer at a temperature of 50 ℃ for 24 hours. The concentration of Li_2_S_6_ achieved in the solution was 0.25 M.

*Synthesis of sulfur/carbon composite.* The typical melt-diffusion method prepared the sulfur/carbon black (Super-P/S) composite. The Super-P and sulfur powder with a weight ratio 3:7 were grounded homogenously, sealed in an Ar-filled stainless steel vessel, and then heated at 155 °C for 12 h.

**Material Characterizations**

Powder X-ray diffraction (PXRD) patterns of all samples were collected by a Rigaku Smartlab 9000W diffractometer with Cu Kα radiation (λ = 0.15418 nm) working at 40 KV and 200mA. Scanning electron microscope (SEM) of all samples was obtained using a field emission scanning electron microscope (FESEM, JEOL JSM-7500FA). All samples' X-ray photoelectron spectra (XPS) patterns were taken on a Thermo ESCALAB 250 spectrometer with a monochromatic Al Kα as the excitation source. A UV-Vis Spectrophotometer (Jasco V-770) (UV-Vis_Jasco) obtained UV-Vis spectra. Raman spectra were collected from a WITec Alpha300R confocal Raman microscope (excitation wavelength 532 nm, grating 600 g/mm).

**Electrochemical Measurements**
Take an 11.9 μL Li_2_S_6_ catholyte and drop it on a circular carbon cloth electrode with a diameter of 12 mm to make a Li_2_S_6_ electrode. The amount of active material in terms of S mass is 0.5 mg/cm^2^. The S_8_ electrode was prepared by mixing sulfur/carbon composite, acetylene black, and polyvinylidene fluoride (PVDF) binder in a weight ratio of 7:2:1 in N-methyl-2-pyrrolidone (NMP). The resulting homogeneous slurry was coated onto the carbon cloth (diameter 12mm) and then dried at 60 ^o^C for 24 h. The total mass loading of sulfur is ~0.5 mg cm^-2^. Coin-type (CR2032) cells were assembled in an Ar-filled glove box (Super 1220/750, Mikrouna), and H_2_O and O_2_ levels were less than 0.1 ppm. The potassium metal sheet was the counter electrode. The electrolyte contained 5 M potassium bis (trifluoromethane sulfonyl) imide (KTFSI) in DEGDME as the electrolyte additive. The amount of electrolyte added to every cell is 150 μL, and the E/S ratio is 300 μL mg^-1^. Charge-discharge tests were performed at 0.5−3.0 V on a Land CT2001A battery test system (Wuhan, China). CV was measured on a Bio-Logic EC-LAB (VMP-300) electrochemical workstation between 0.5 and 3.0 V at a scan rate of 0.1 mV s^−1^. EIS was obtained in a frequency range from 0.01 Hz to 100 kHz.

**Computational Methods**

The first-principles calculations were conducted by the Vienna Ab-initio Simulation Package (VASP)^[1]^ software with the Projected Augmented Wave (PAW) pseudopotential method. The Perdew-Burke-Enzerhof (PBE)^[2]^ formulation described the electron exchange function under the Generalized Gradient Approximation (GGA)^[3]^. The kinetic energy cutoff for the electronic wavefunction was 520 eV. A Gamma-centered 2 × 2 × 1 k-points grid was used for the geometry optimization and 4 × 3 × 2 k-points for the electronic structure calculations. A vacuum thickness of 20 Å in the z-direction was built to avoid the interactions between periodically repeated images. The van der Waals (vdW) interactions were included using the DFT-D3 method with Becke-Johnson damping function^[4, 5]^.

The Gibbs free energy was obtained as

$$G=E+ZPE - TS + eU$$

where *E* is the DFT ground state total energy, *ZPE*-*TS* is the free energy corrections calculated over the vibration frequencies according to the harmonic approximation, and *U* is the applied potential.


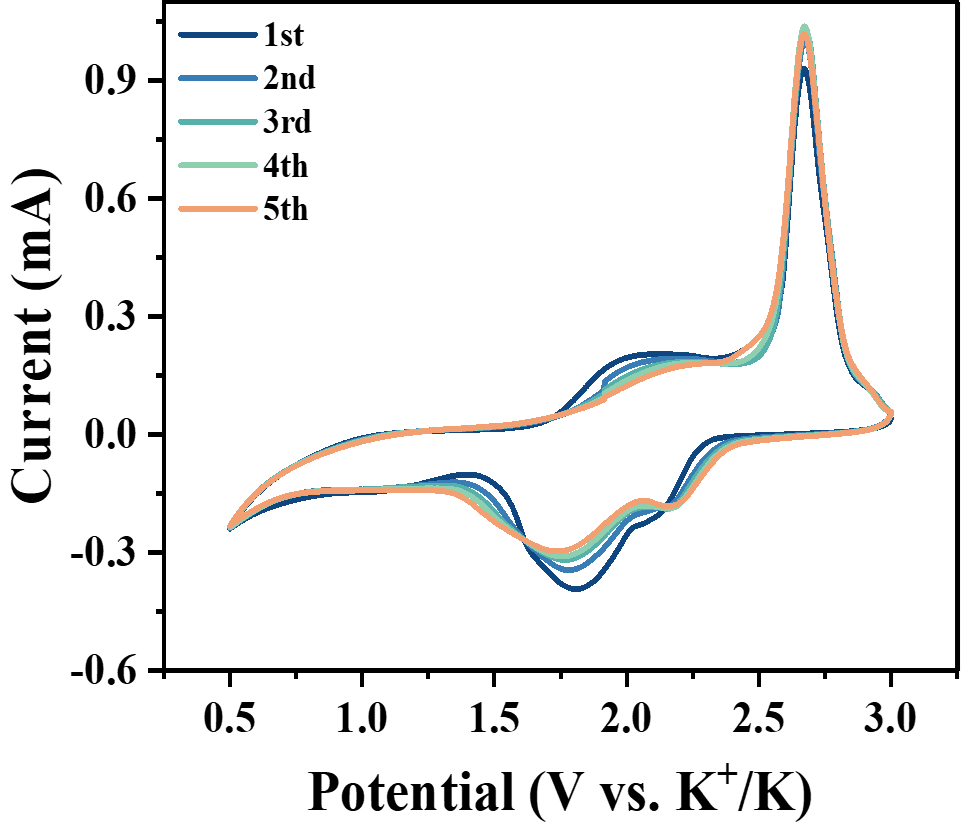


**Figure S1**. 1st-5th cycles CV curves of Li_2_S_6_-based K-S Battery


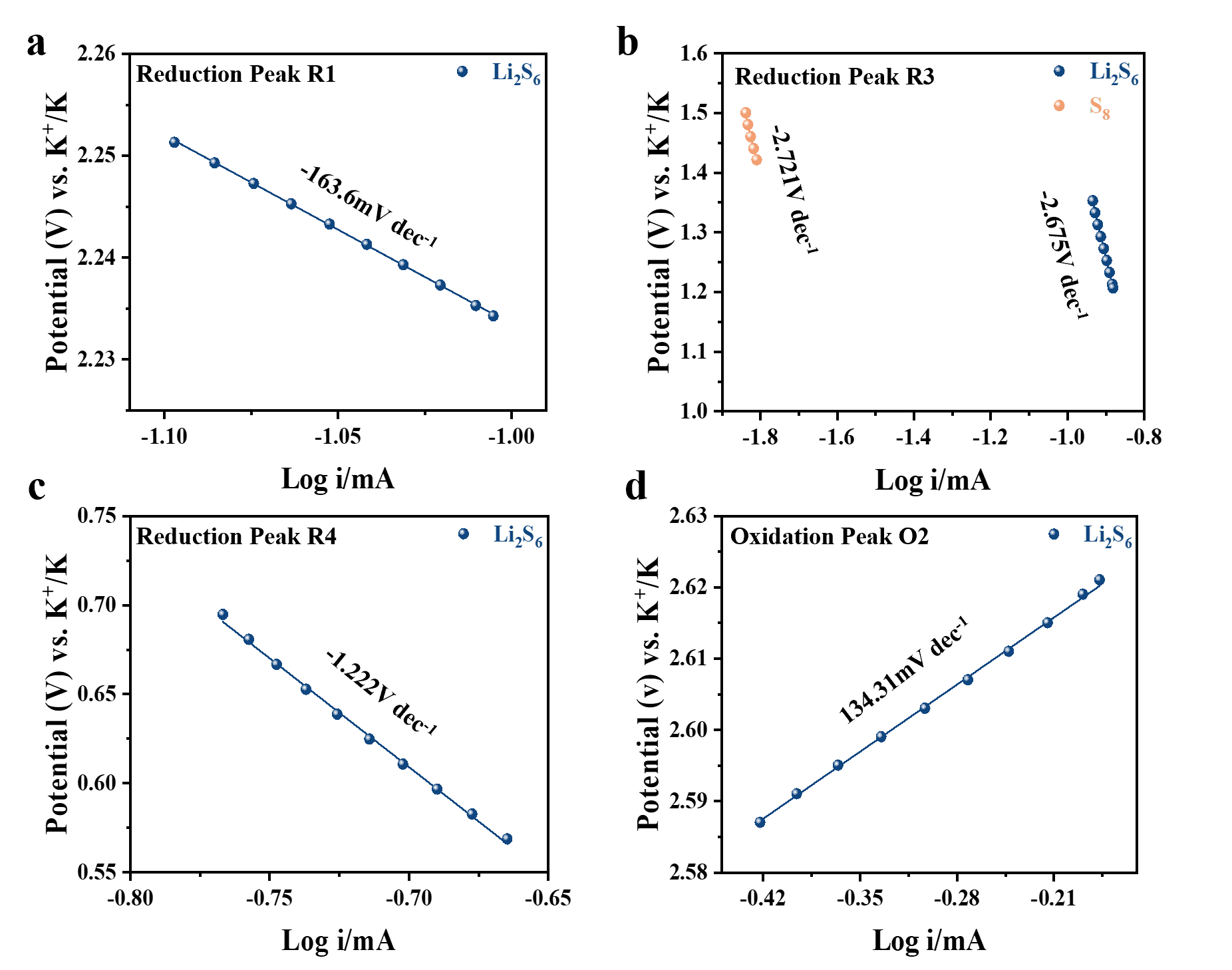


**Figure S2**. Tafel plots of other redox peaks: a) R1, b) R3, c) R4, d) O2.

**Figure S3**. Cyclic performance of carbon cloth electrode (calculated based on 0.5 mg of active material, for easier comparison of the carbon cloth's impact during K-S battery testing).


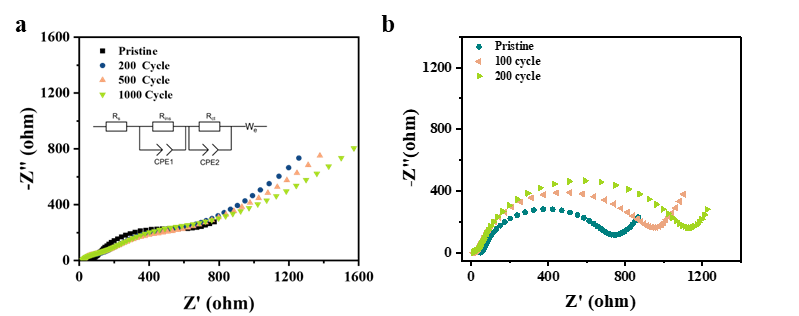


**Figure S4**. Nyquist plots of pristine, 200 cycles, 500 cycles, and 1000 cycles, with equivalent circuit for a) Li_2_S_6_ and b)S_8_.**Figure S5**. R_ins_'s comparison of Li_2_S_6_-based K-S battery 200 cycles, 500 cycles, and 1000 cycles.


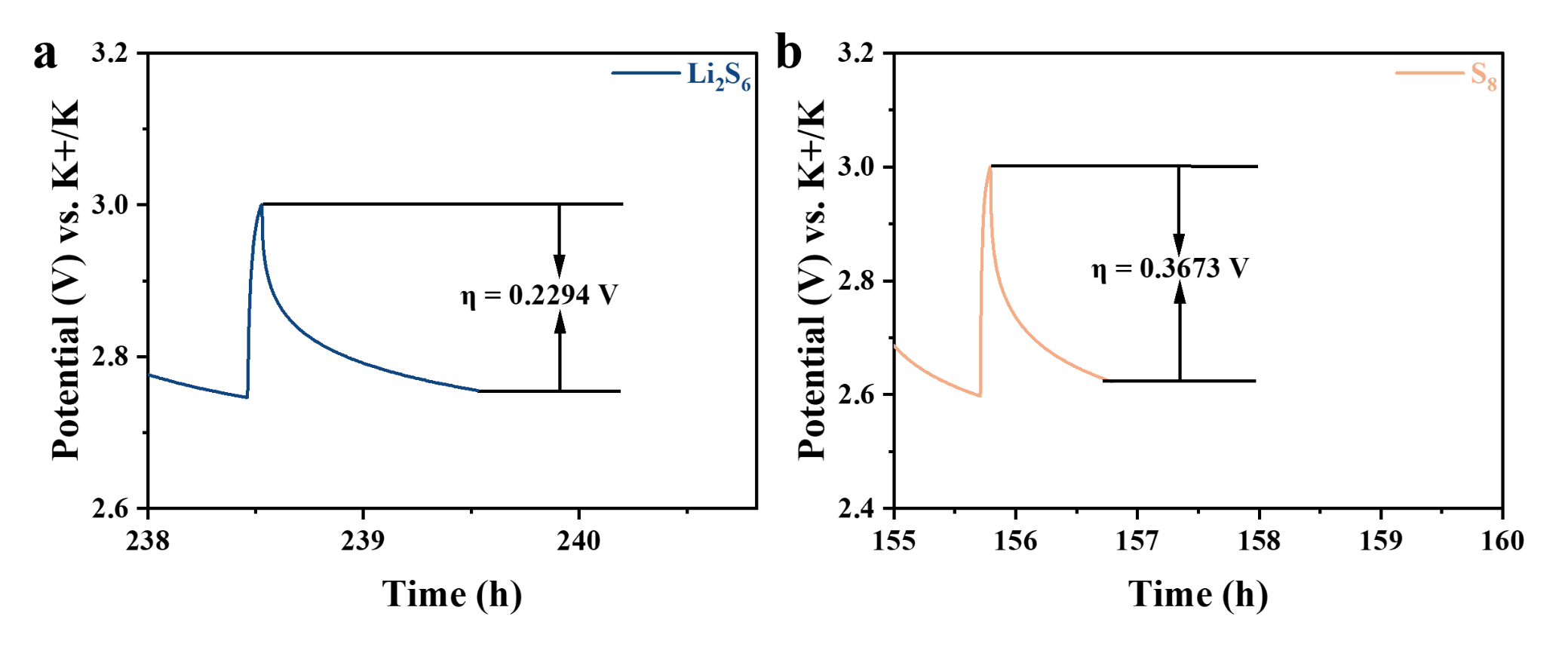


**Figure S6**. Locally amplified GITT final charge voltage profiles and overpotential of a) Li_2_S_6_ and b) S_8_

**Reaction Resistance Calculation**

**Formula S1**

$$R_{r}= \frac{\text{η}}{I}$$

R_r_: Discharge and charge reaction resistance

η: Overpotential (gain from GITT)

I: Current of the GITT test

**Figure S7.** CV curves of Li_2_S_6_ symmetrical cells.


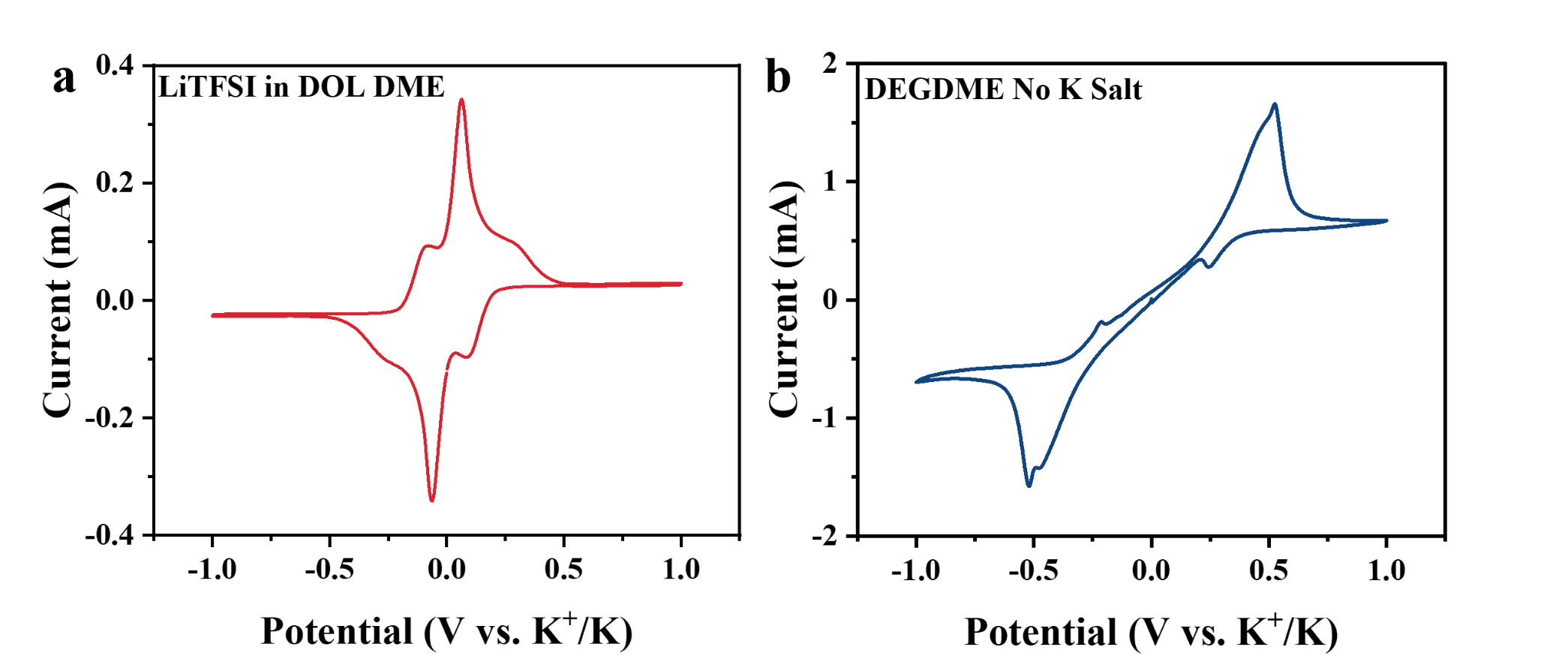


**Figure S8**. a) CV curves of the Li_2_S_6_ electrode symmetric cell with Li-S electrolyte and b) pure DEGDME.

**Figure S9**. The cycling performance of different E/S ratio of Li_2_S_6_ based K-S batteries.


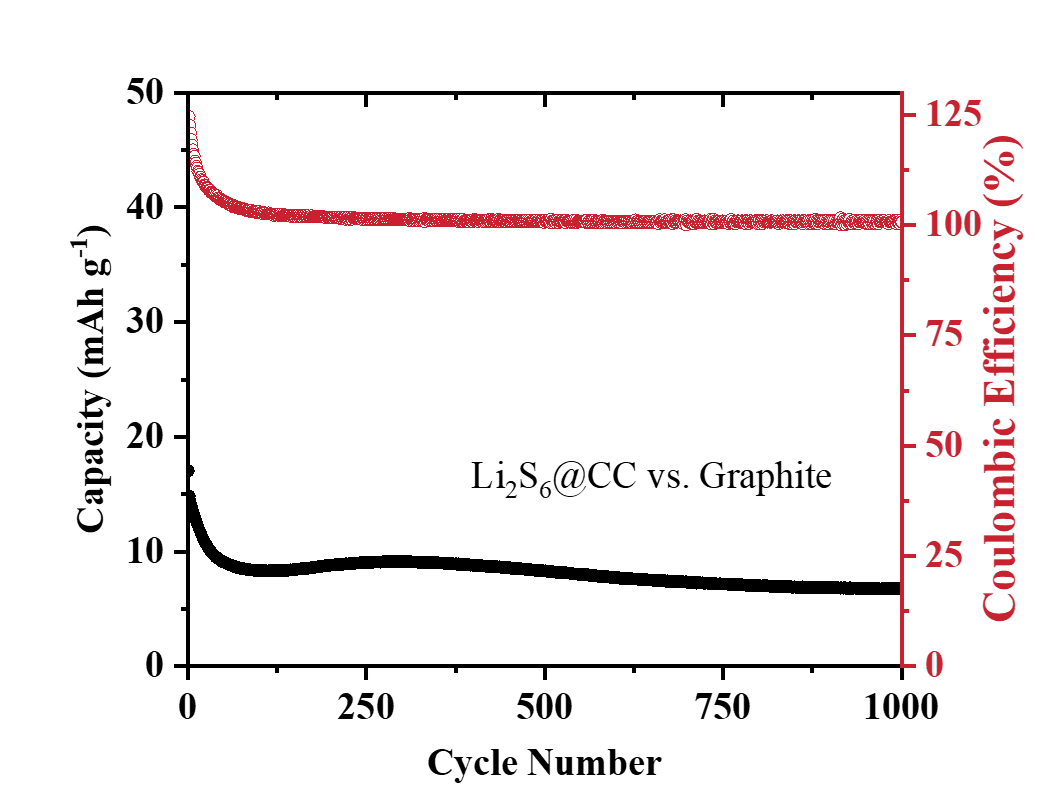
**Figure S10**. The cycling performance of Li_2_S_6_ electrode vs. Graphite Anode with same 5M KTFSI DEGDME electrolyte.


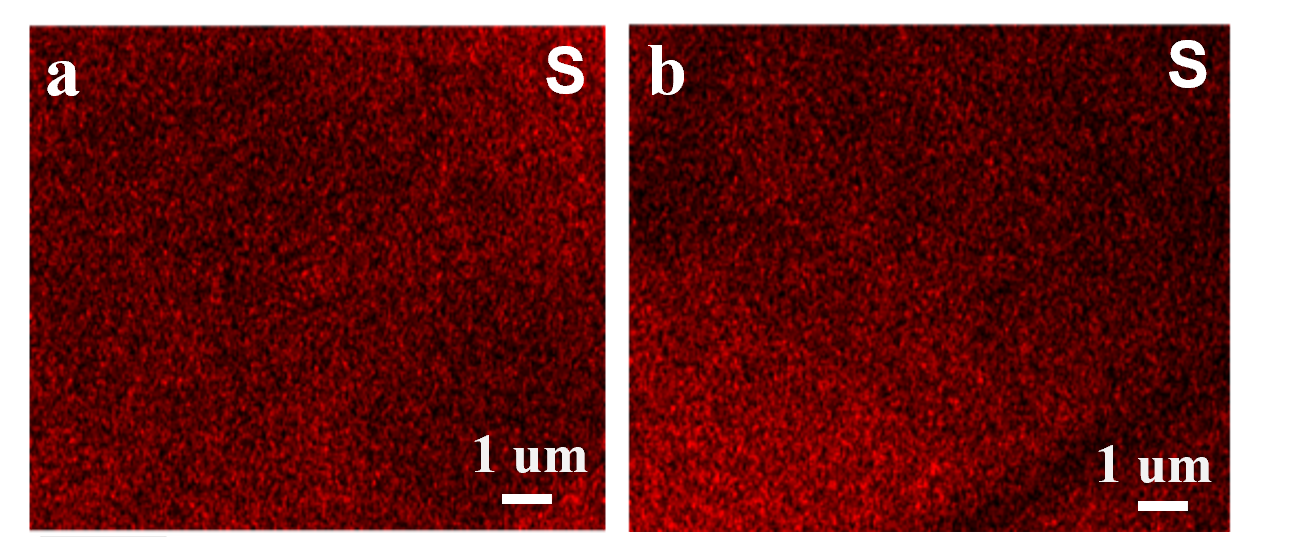


**Figure S11**. The Energy Dispersive X-ray Spectroscopy (EDX) sulfur element mapping of a) pristine and b) after 1000 cycles of Li_2_S_6_ electrode.


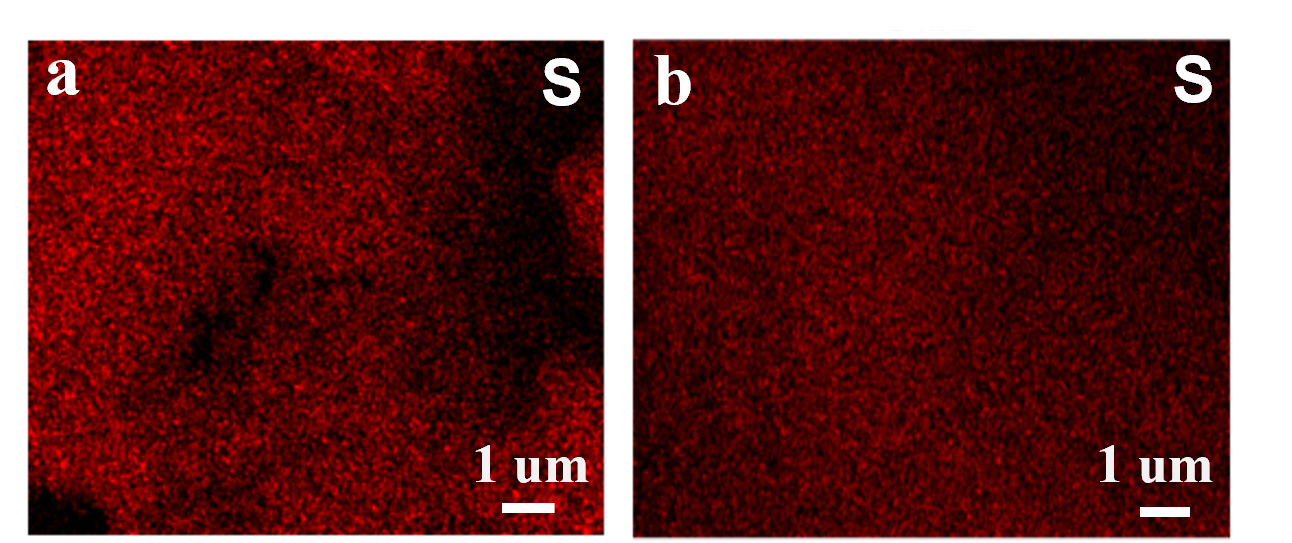


**Figure S12**. The Energy Dispersive X-ray Spectroscopy (EDX) sulfur element mapping of a) pristine and b) after 200 cycles of S_8_ electrode.

**
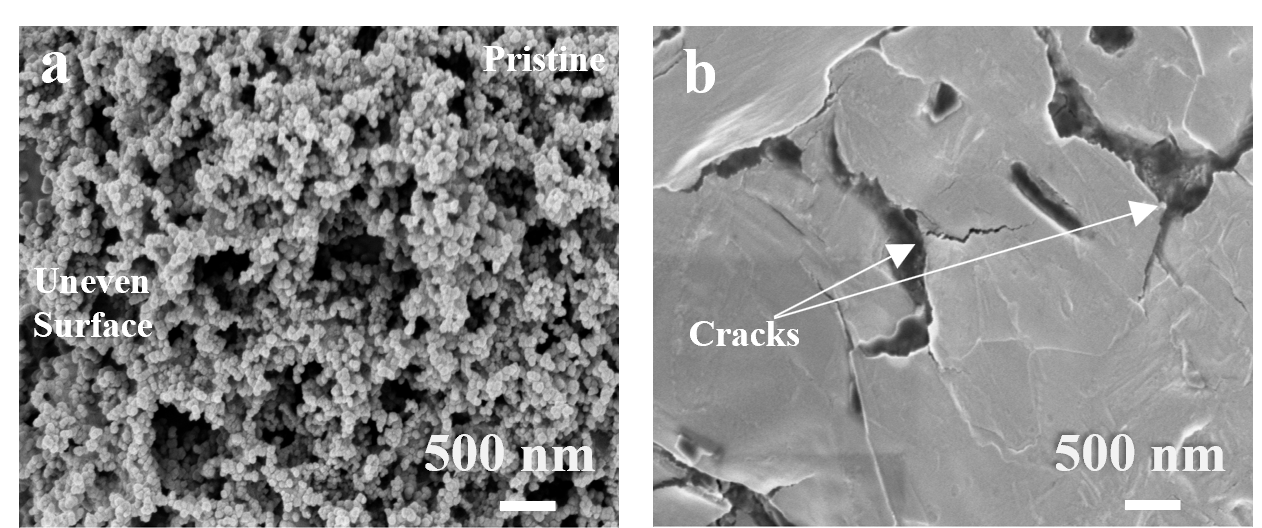
**

**Figure S13**. SEM images of electrode surfaces: a) pristine and f) after 1000 cycles of S_8_ electrode


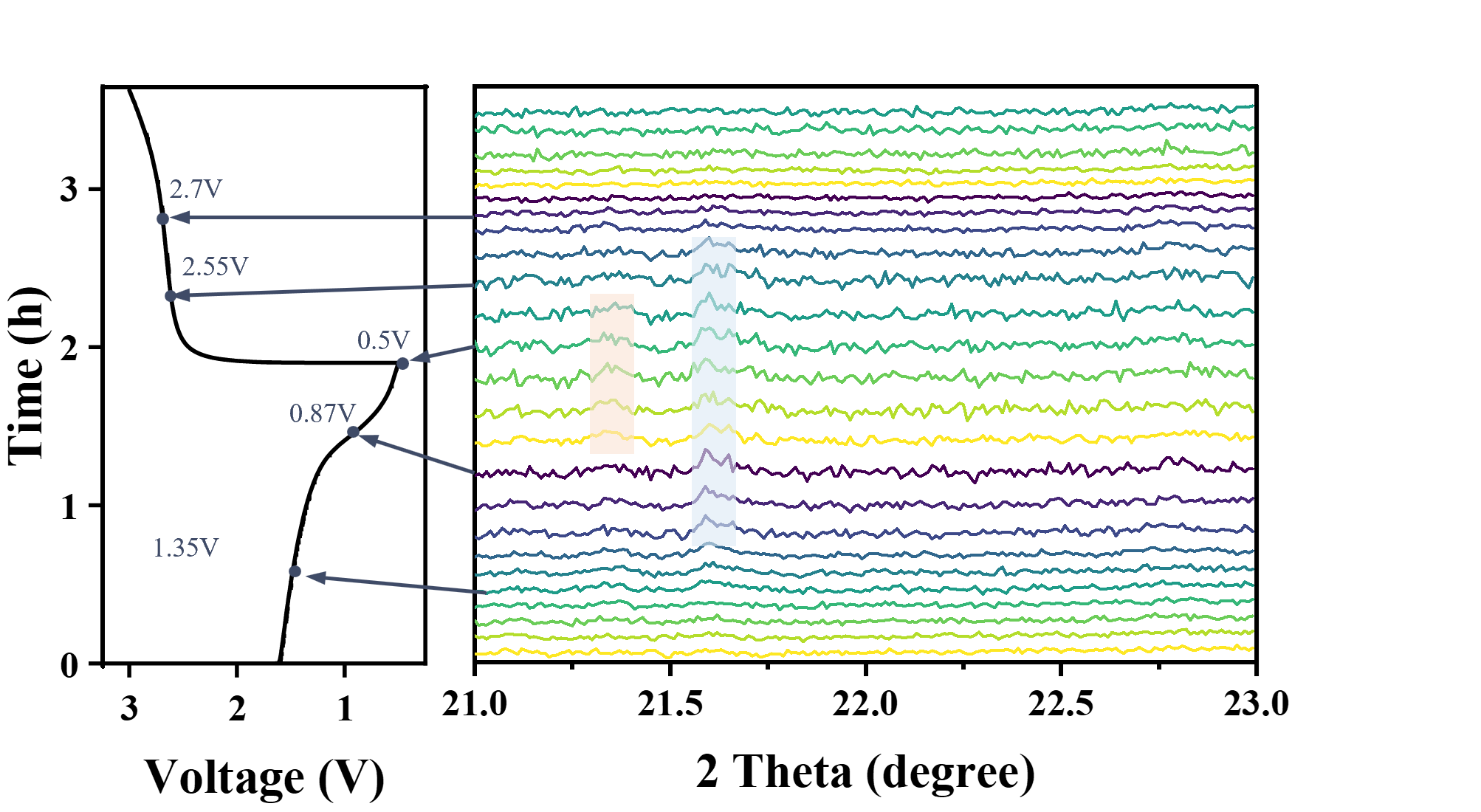
**Figure S14**. Original data of *Operando* XRD patterns for Li_2_S_6_ electrode.

**Figure S15**. XPS test of the cathode at the state of charge to 3.0 V.


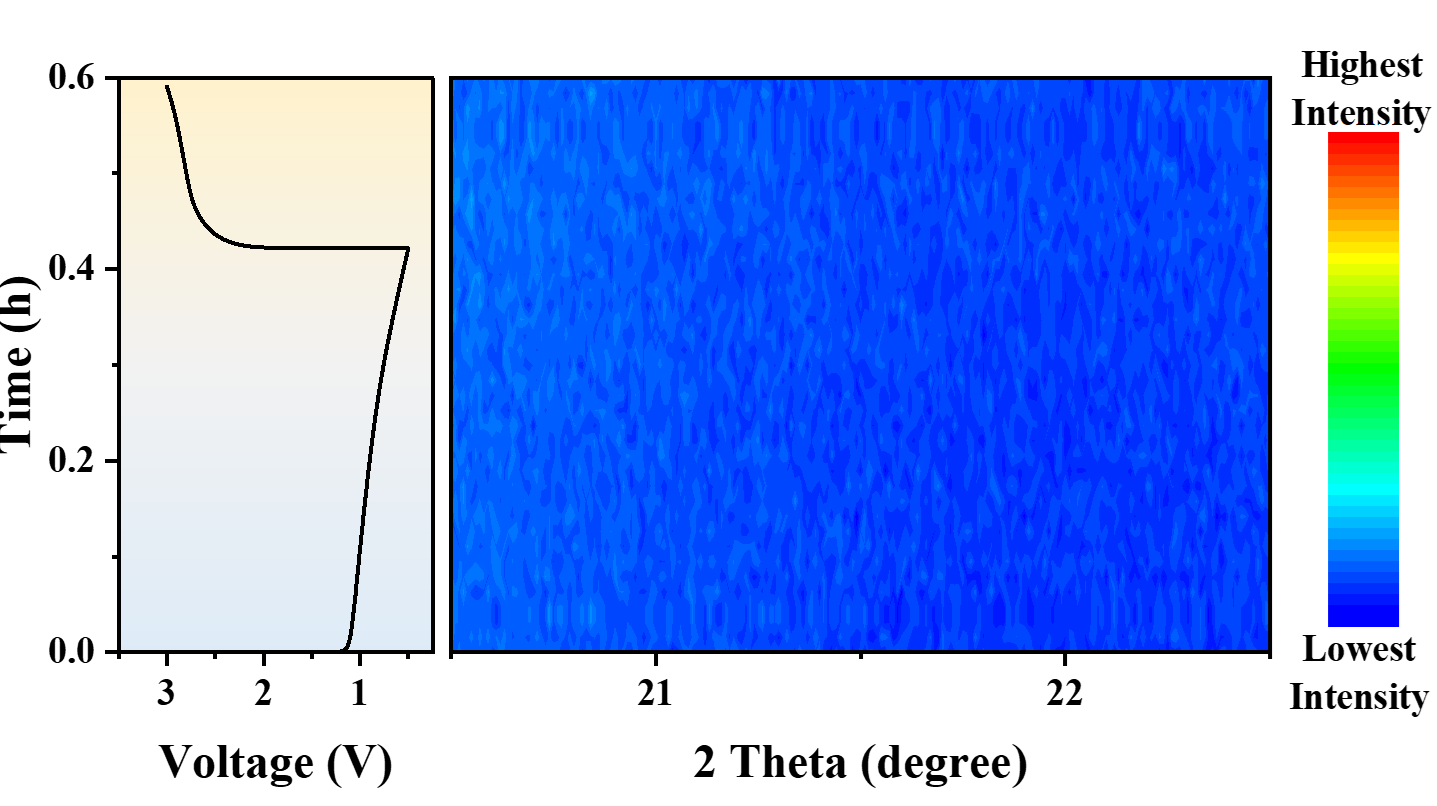


**Figure S16**. Original data of *Operando* XRD patterns for S_8_ electrode.


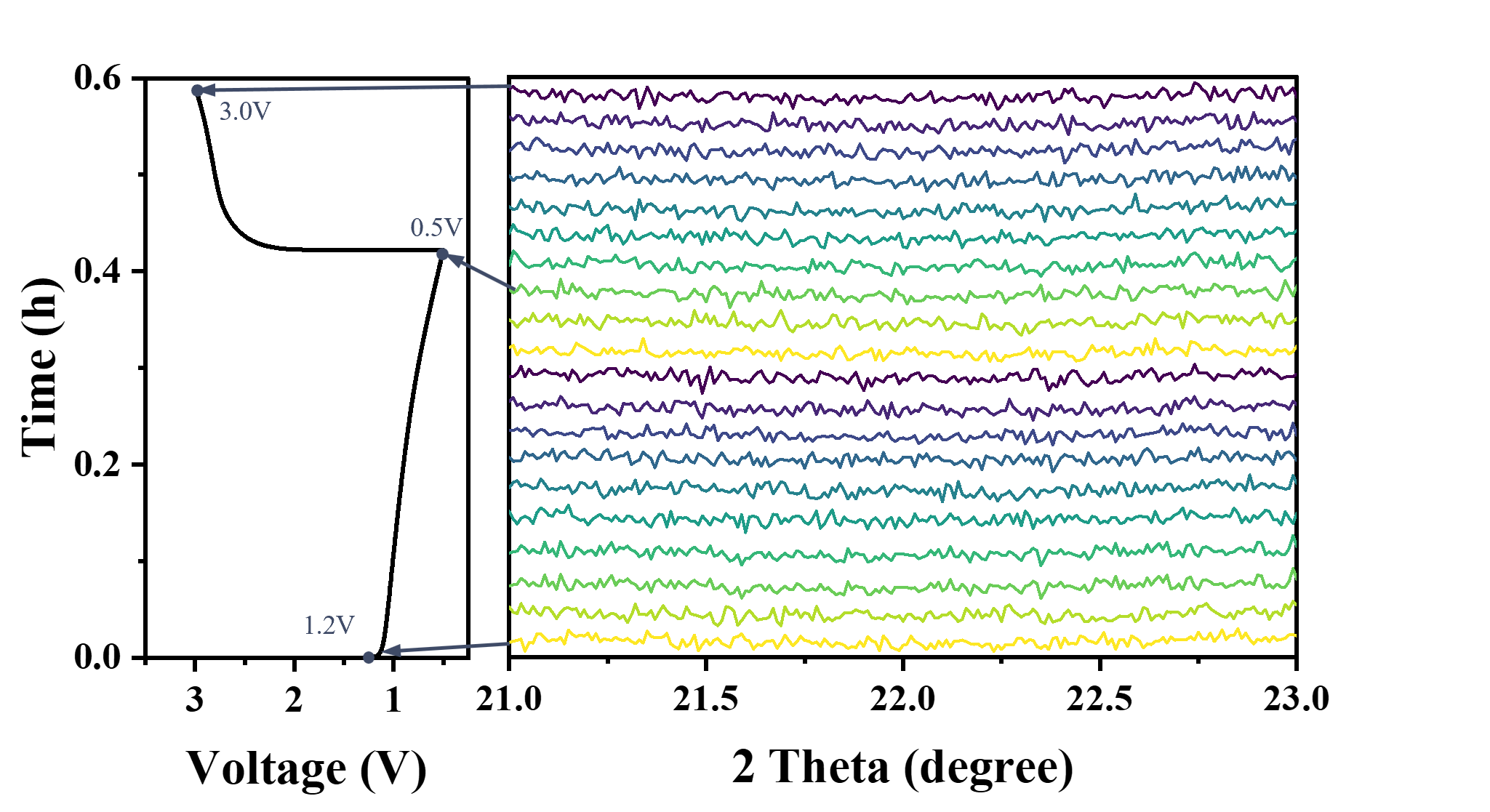
**Figure S17**. Original data of *Operando* XRD patterns for S_8_ electrode.


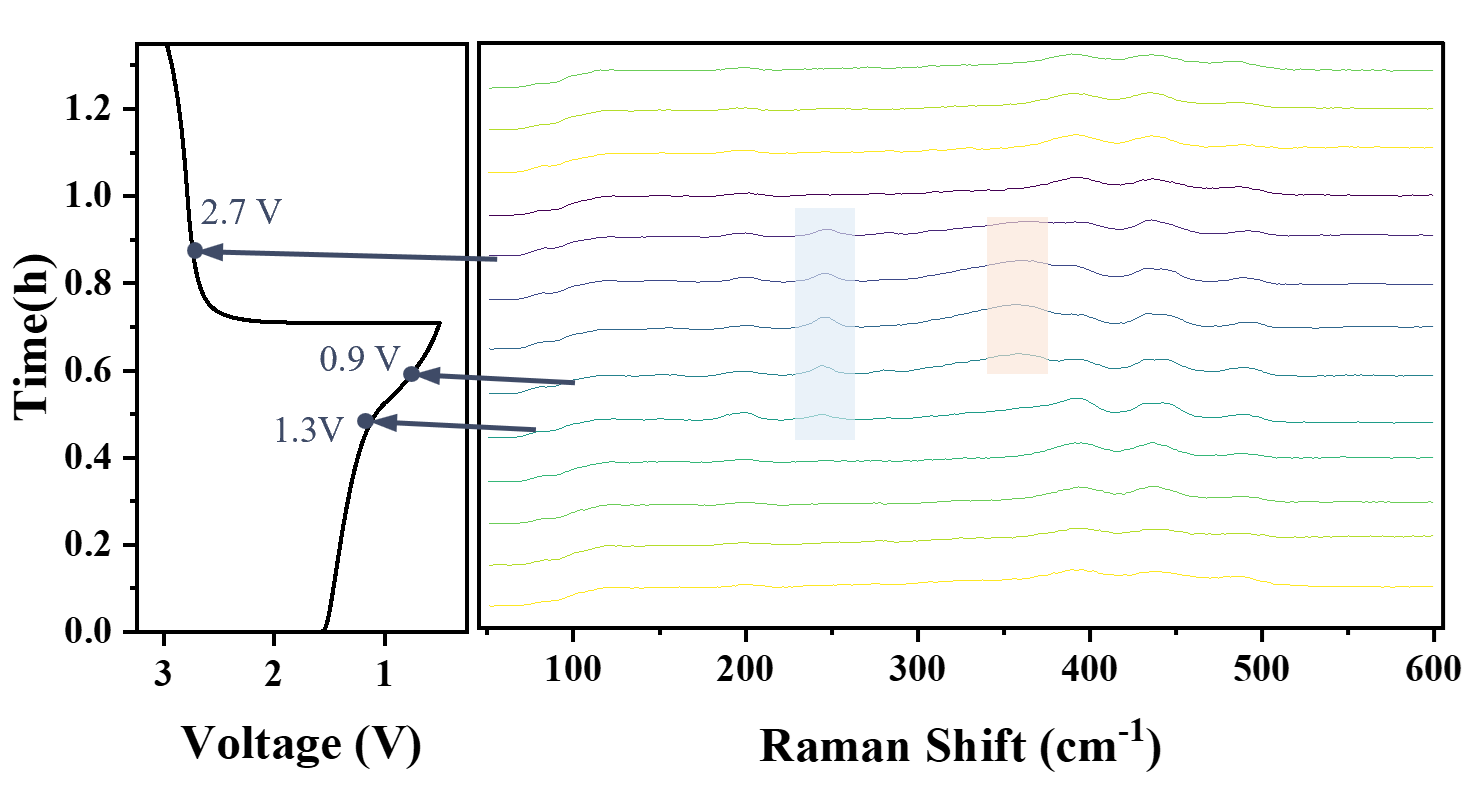


**Figure S18**. Original data of *Operando* Raman patterns for Li_2_S_6_ electrode.


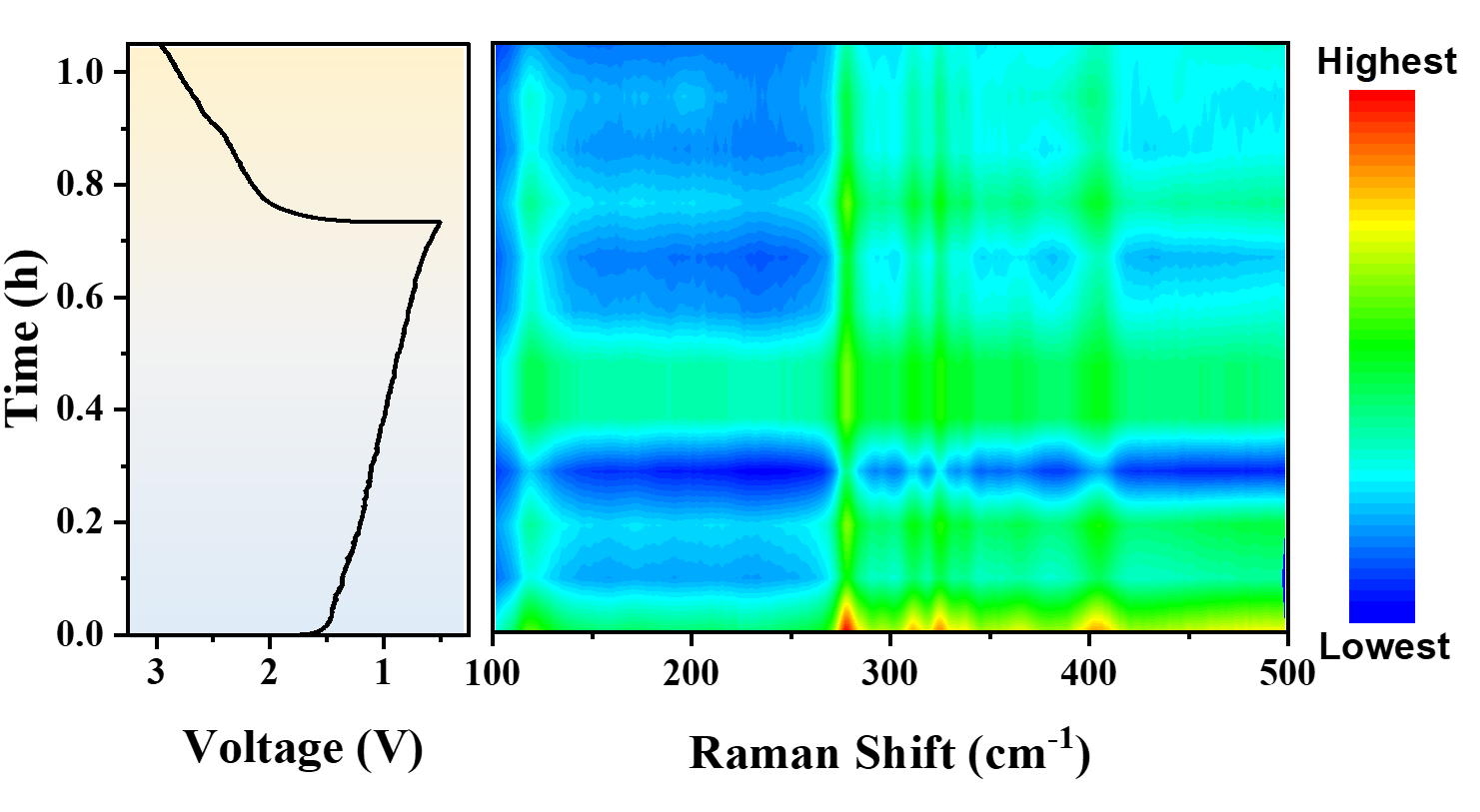


**Figure S19**. *Operando* Raman patterns for S_8_ electrode.


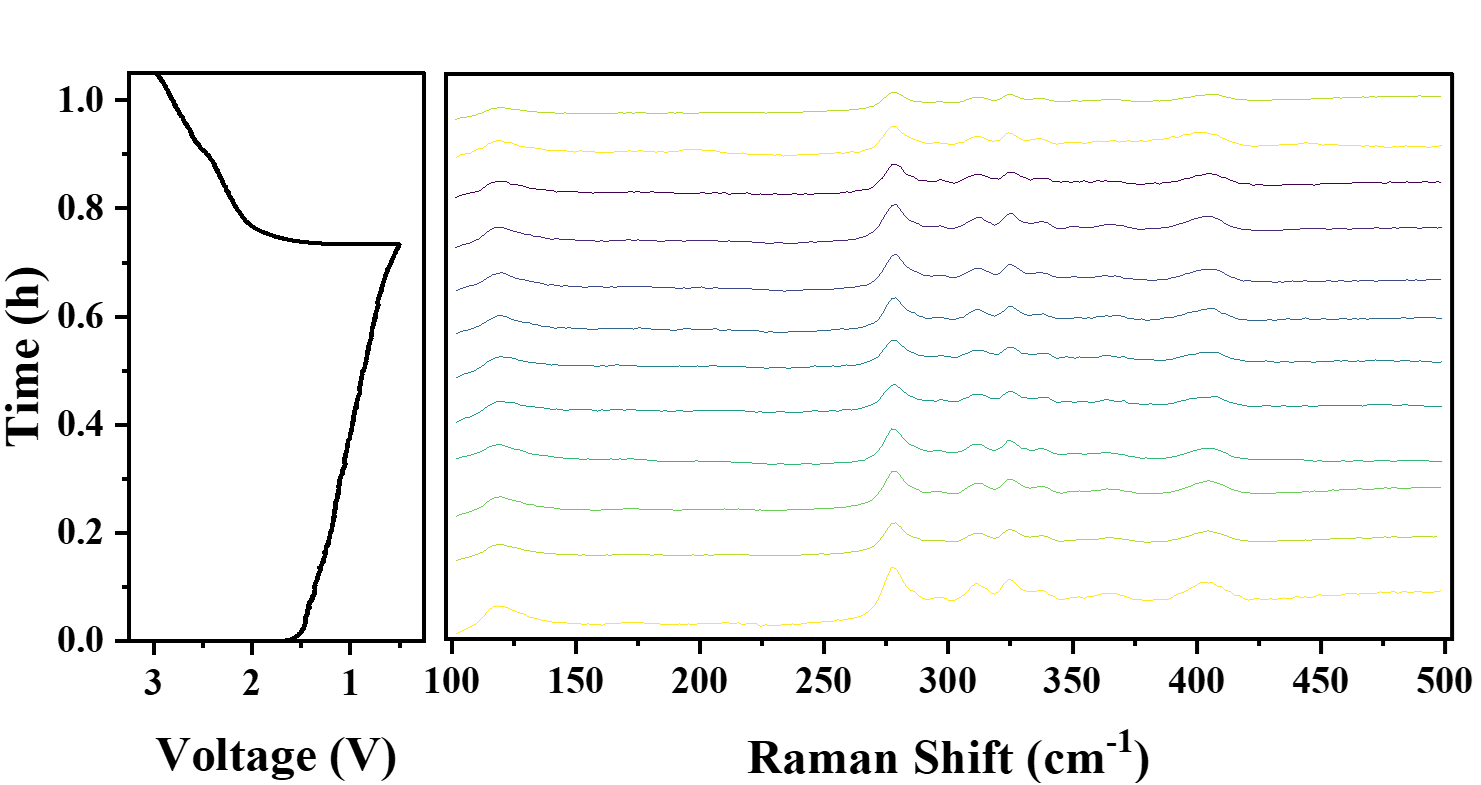


**Figure S20**. Original data of *Operando* Raman patterns for S_8_ electrode.


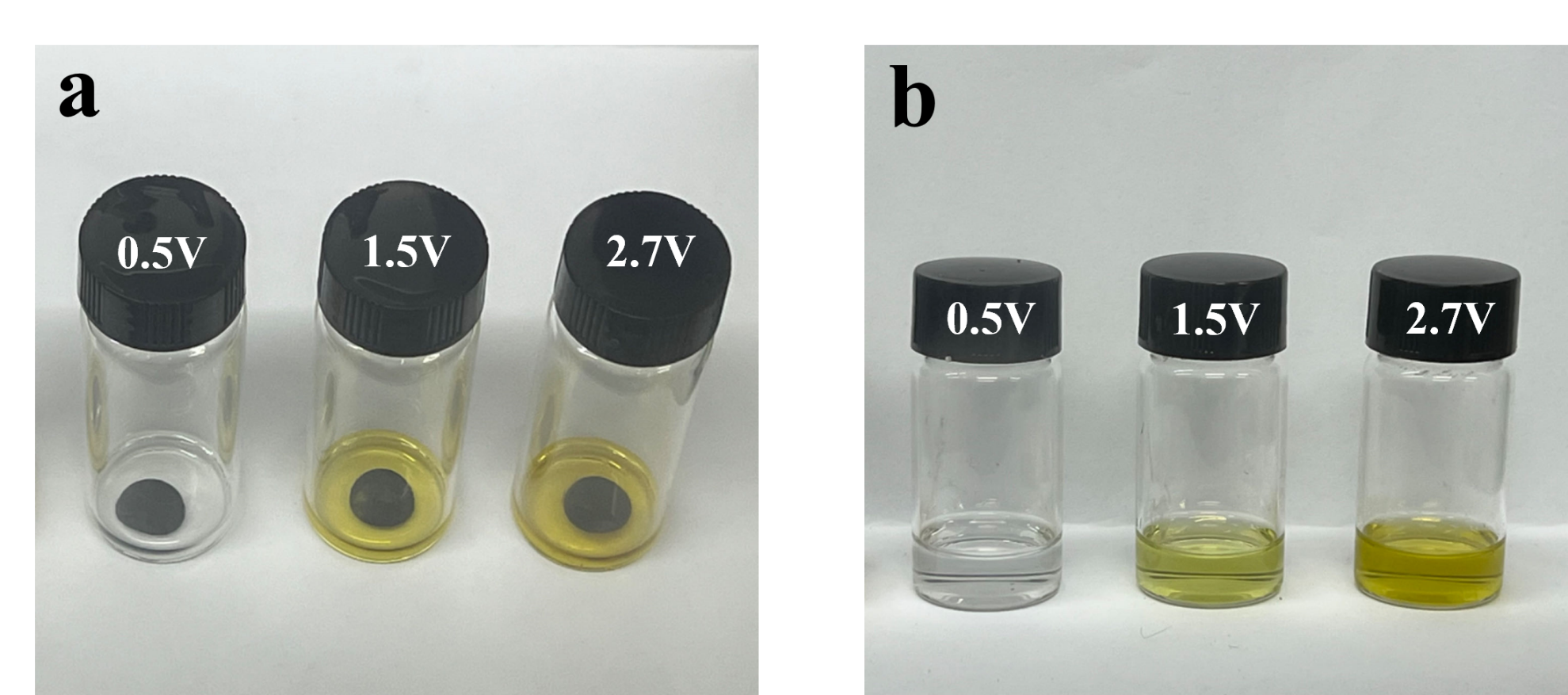
**Figure S21**. a) The optical images of the S cathodes at discharging stages of 0.5V, 1.5V, and charge stage of 2.7 V are immersed in dimethyl ether (DME), respectively. b) The dilution results of the supernatant.


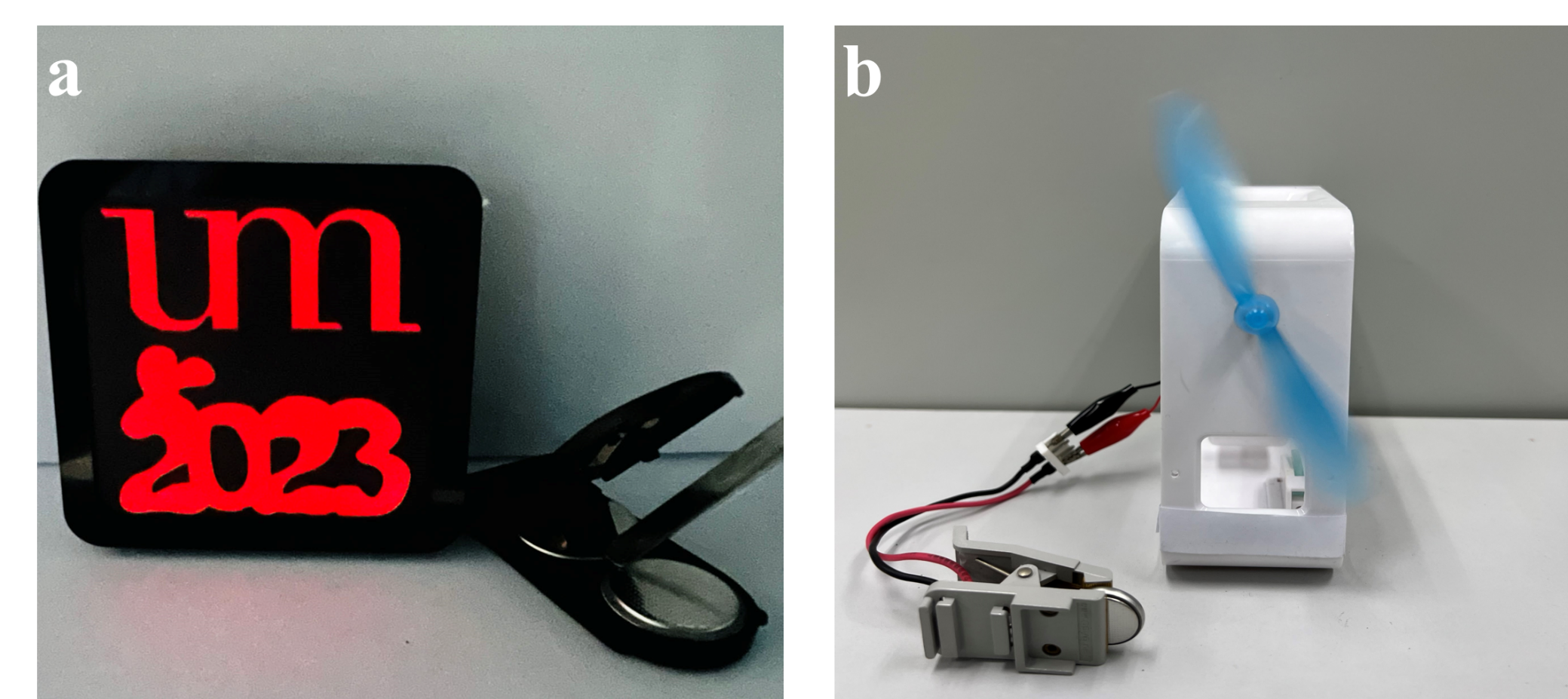


**Figure S22.** a) Optical images of coin cells lighting LED’s UM 2023 logo and b) powering the fan.

**Table S1.** Comparison of the electrochemical performances of various catalysts between this work and other reported studies.

| Materials | Electrolyte | Current Collector | Working Current | Cycle Number | Retained Discharge Capacity | Ref. |
| --- | --- | --- | --- | --- | --- | --- |
| Catholyte: Li_2_S_6_ | 5 M KTFSI in DEGDME | Carbon Cloth | 0.5 C | 1000 | 294 mAh g^-1^ | This Work |
| SPAN | 0.8 M KPF_6_ in EC/DEC | - | 0.5 C | 100 | 147 mAh g^-1^ | [6] |
| Covalent CCS | 1 M KSO_3_CF_3_ in EC/DEC | Al foil | 150 mA g^-1^ | 300 | 253 mAh g^-1^ | [7] |
| EAMC12 (C/S composites) | 0.8 M KPF_6_ in EC/DEC | Al foil | 0.2 C | 500 | 281.2 mAh g^−1^ | [8] |
| K–Na/SPAN | 1 M KPF_6_ in EC/DMC/EMC | Al foil | 35 mA g^-1^ | 100 | 140 mAh g^−1^ | [9] |
| Poly(S_4_-TABQ) | 1 M KTFSI in TEGDME | Al foil | 0.2 C | 200 | 119 mAh g^−1^ | [10] |
| CNT/S composite | 3 M KFSI in DME | - | 0.05 C | 200 | 135 mAh g^−1^ | [11] |
| S@SA-NC | 0.8 M KPF_6_ in EC/DEC | Cu foil | 0.5C | 200 | 371 mAh g^−1^ | [12] |
| Catholyte: Sulfur + K_2_S_x_ | 0.5 M KTFSI in DEGDME | - | 0.1 C | 20 | 310 mAh g^−1^ | [13] |
| S-N-Co_s_-C | 0.8 M KPF_6_ in EC/DEC | Al foil | 50 mA g^-1^ | 50 | 453 mAh g^−1^ | [14] |
| S/Cu-N_4_ | 0.8 M KPF_6_ in EC/DEC | Cu foil | 1 C | 100 | 480 mAh g^−1^ | [15] |

**References**

1. Kresse, G. and J. Furthmüller, *Efficient iterative schemes for ab initio total-energy calculations using a plane-wave basis set.* Physical Review B, 1996. **54**(16): p. 11169-11186.

2. Perdew, J.P., K. Burke, and M. Ernzerhof, *Generalized Gradient Approximation Made Simple.* Physical Review Letters, 1996. **77**(18): p. 3865-3868.

3. Perdew, J.P. and Y. Wang, *Accurate and simple analytic representation of the electron-gas correlation energy.* Physical Review B, 1992. **45**(23): p. 13244-13249.

4. Grimme, S., S. Ehrlich, and L.J.J.o.c.c. Goerigk, *Effect of the damping function in dispersion corrected density functional theory.* 2011. **32**(7): p. 1456-1465.

5. Grimme, S., et al., *A consistent and accurate ab initio parametrization of density functional dispersion correction (DFT-D) for the 94 elements H-Pu.* The Journal of Chemical Physics, 2010. **132**(15).

6. Liu, Y., et al., *Sulfur nanocomposite as a positive electrode material for rechargeable potassium–sulfur batteries.* Chemical Communications, 2018. **54**(18): p. 2288-2291.

7. Ma, R., et al., *Confined and covalent sulfur for stable room temperature potassium-sulfur battery.* Electrochimica Acta, 2019. **293**: p. 191-198.

8. Hu, L., et al., *A superficial sulfur interfacial control strategy for the fabrication of a sulfur/carbon composite for potassium–sulfur batteries.* Chemical Communications, 2021. **57**(12): p. 1490-1493.

9. Zhang, Y., et al., *A novel rechargeable potassium–sulfur battery based on liquid alloy anode.* Materials Letters, 2019. **242**: p. 5-8.

10. Zhang, L., et al., *Tuning the Linkers in Polymer-Based Cathodes to Realize High Sulfur Content and High-Performance Potassium–Sulfur Batteries.* The Journal of Physical Chemistry C, 2021. **125**(34): p. 18604-18613.

11. Yuan, X., et al., *High-Performance Stable Potassium–Sulfur Batteries Enabled by Free-Standing CNT Film-Based Composite Cathodes.* Journal of Electronic Materials, 2021. **50**(6): p. 3037-3042.

12. Ye, C., et al., *Catalytic Oxidation of K(2)S via Atomic Co and Pyridinic N Synergy in Potassium-Sulfur Batteries.* J Am Chem Soc, 2021. **143**(41): p. 16902-16907.

13. Hwang, J.-Y., et al., *Toward High-Safety Potassium–Sulfur Batteries Using a Potassium Polysulfide Catholyte and Metal-Free Anode.* ACS Energy Letters, 2018. **3**(3): p. 540-541.

14. Ge, X., et al., *Metal–Organic Framework-Derived Nitrogen-Doped Cobalt Nanocluster Inlaid Porous Carbon as High-Efficiency Catalyst for Advanced Potassium–Sulfur Batteries.* ACS Nano, 2020. **14**(11): p. 16022-16035.

15. Ye, C., et al., *Reducing Overpotential of Solid-State Sulfide Conversion in Potassium-Sulfur Batteries.* Angewandte Chemie International Edition, 2023. **62**(22): p. e202301681.
